# Repeated Transcranial Direct Current Stimulation Induces Behavioral, Metabolic and Neurochemical Effects in Rats on High-Calorie Diet

**DOI:** 10.3389/fnbeh.2017.00262

**Published:** 2018-01-15

**Authors:** Agata Ziomber, Eugeniusz Rokita, Jolanta Kaszuba-Zwoinska, Irena Romańska, Jerzy Michaluk, Lucyna Antkiewicz-Michaluk

**Affiliations:** ^1^Chair of Pathophysiology, Faculty of Medicine, Jagiellonian University Medical College, Krakow, Poland; ^2^Chair of Physiology, Department of Biophysics, Faculty of Medicine, Jagiellonian University Medical College, Krakow, Poland; ^3^Department of Neurochemistry, Institute of Pharmacology Polish Academy of Sciences, Krakow, Poland

**Keywords:** transcranial direct current stimulation, feeding behavior, high-calorie diet, obesity, brain monoamines

## Abstract

Due to its high prevalence, obesity is considered an epidemic, which stimulated research on non-invasive methods to reduce excess body fat. Transcranial direct current stimulation (tDCS) is a non-invasive technique used to modulate the activity of cerebral cortex, which has already found increasing interest in medicine as a promising methodology. The aim of this study was to analyze the impact of tDCS on feeding behavior, metabolic abnormalities and neurotransmitters in certain brain areas involved in appetite control of obese rats. The male Wistar rats were divided into five subgroups depending on consumed diet effect (lean, obese) and tDCS type (anodal, cathodal, sham, and no stimulation). Two 10-min daily sessions of tDCS for 8 consecutive days of the study were applied. Rats subjected to active tDCS (anodal right or cathodal left of the prefrontal cortex) had reduced appetite and showed lesser body weight gain than the animals subjected to sham procedure or those receiving no stimulation at all. Furthermore, tDCS contributed to reduction of epididymal fat pads and to a decrease in blood concentration of leptin. Neurochemical examination revealed that tDCS modulated serotonin pathways of the reward-related brain areas and contributed to a significant decrease in the density of D2 but not D1 dopamine receptors in the dorsal striatum, recorded 5 h after the last stimulation. No significant effect of tDCS on dopamine and it's metabolites in examined brain regions was observed. It seems that the hypothalamus was not affected by tDCS application as no changes in measured neurotransmitters were detected at any examined time point. However, these results do not exclude the possibility of the delayed response of the monoamines in the examined brain areas to tDCS application. Altogether, these findings imply that repeated tDCS of the prefrontal cortex may change feeding behavior of obese rats. Either right anodal or left cathodal tDCS were sufficient to decrease food intake, to reduce body adiposity and to normalize other metabolic anomalies. These beneficial effects can be at least partially explained by changes in serotoninergic and in lesser extent dopaminergic system activity within some brain areas belonging to reward system.

## Introduction

### Regulation of energy metabolism

Due to its high prevalence, obesity is currently considered an epidemic. Owing multiple documented interactions between the brain, gut and adipose tissue, obesity is nowadays recognized as a neurological disease, rather than a simple metabolic disorder. Hypothalamus is a key region of the brain, responsible for homeostatic control of appetite. Hypothalamus integrates peripheral signaling from the gut, adipose tissue and other areas involved in energy metabolism, with information from the brainstem and higher cortical centers, such as reward-related regions (Benarroch, 2010). Reward-related limbic pathways are involved in non-homeostatic regulation of food intake, and may override normal satiety signals, which may result in hyperphagia and obesity. Corticolimbic pathways terminate in the striatum (STR), nucleus accumbens (NAc), and orbitofrontal cortex (PC). Information about the taste, appearance, smell and texture of foods is sent to orbitofrontal cortex, a somatosensory structure playing a role in reward-related feeding (Rolls, 2011). Limbic and paralimbic cortex (both responsible for regulation of emotions) are primarily activated during hunger, whereas activation of prefrontal cortex results in satiety (Tataranni et al., 1999). Cerebral mechanisms that underlie eating behaviors and may eventually result in pathological overeating and obesity, are still poorly understood. Dopamine, a neurotransmitter modulating rewarding properties of foods, especially those rich in sugars or fat, is likely to be involved. The fact that consumption of a palatable food stimulates the release of dopamine in dopaminergic mesolimbic reward system (nucleus accumbens) and nigrostriatal system (dorsal striatum) (Small et al., 2003) points to an important role of this neurotransmitter in reward-related food intake. A diet-induced reward deficit in overweight rats may reflect a counter-adaptive decrease in baseline sensitivity of their brain reward systems, preventing overstimulation by palatable foods. Downregulation of striatal dopamine receptors D_2_ is a notable neuroadaptive response to overconsumption of a palatable food. Indeed, a decrease in striatal D_2_ receptor density was demonstrated both in overweight humans and rodents (Wang et al., 2001; Huang et al., 2006). On the other hand, serotonin is a powerful inhibitor of feeding. Previous studies with pharmacological modulators of serotonergic system demonstrated that increased serotonin stimulation results in a decrease in food intake and reduction of body weight in both animals and humans (McTavish and Heel, 1992; Simansky, 1996).

### Transcranial direct current stimulation (tDCS)

Currently, treatment options in obesity are limited, which stimulated research on novel non-invasive therapies for this condition. tDCS is a promising experimental approach that uses constant low current delivered directly to the brain via transcranial electrodes. This safe, well-tolerated, simple and inexpensive procedure resulting in modulation of cortical excitability, has already found an application in treatment of neuropsychiatric and neurological disorders, such as epilepsy, Parkinson's disease, Alzheimer's disease, migraine, major depression and post-stroke complications (Medeiros et al., 2012). Recently, tDCS has been also proposed as a method to decrease craving for food (Ljubisavljevic et al., 2016; Macedo et al., 2016). Despite multiple efforts in this matter, still little is known about the mechanisms mediating clinical effects of tDCS. According to one of the most widely accepted hypotheses, anodal stimulation results in neuronal excitability, whereas an opposite effect is exerted by cathodal stimulation (Fritsch et al., 2010; Kabakov et al., 2012). However, biological effects of tDCS, as well as neurotransmitters, neural pathways and neurochemical markers involved in response to the electrostimulation are not completely understood.

The aim of this study was to analyze feeding behaviors, metabolic and neurochemical responses to repeated tDCS in rats maintained on high-calorie diet. Since previous studies demonstrated that both anodal and cathodal tDCS of prefrontal cortex influence appetite (Sauvaget et al., 2015) we applied anodal stimulation to the right prefrontal cortex and cathodal stimulation to the left. The choice of such stimulation protocol was based on a theory that obesity is a consequence of interhemispheric activity imbalance, with right hemispheric hypofunction (Alonso-Alonso and Pascual-Leone, 2007). We assumed that tDCS-mediated excitation of the right prefrontal cortex or inhibition of the left prefrontal cortex may restore the interhemispheric balance. Therefore, we expected that tDCS-exposed animals may show changes in their feeding behaviors, improvement in metabolic parameters and altered neurochemical profiles of the brain regions being involved in energy homeostasis.

## Materials and methods

### Animal housing and the study protocol

The study included 57 male 12–13 week-old Wistar rats, obtained from the animal house at the Faculty of Pharmacy, Jagiellonian University in Krakow. The animals were kept in plastic rectangular cages (cage dimensions: 59 × 38 × 20 cm or 61 × 44 × 22 cm, for 2 or 3 rats in a cage, respectively) in an air-conditioned room with 21–25°C temperature, 55–65% humidity and 12-h to 12-h light to dark cycle. The study was divided into two parts: prestimulation period (from 1st to the 30th day of the experiment) and stimulation period (from 31st to the 37th day of the study). At the beginning of the prestimulation time, 10 days after acclimatization to new conditions, the rats, with mean body weight of 330 g, were randomized to feeding with standard chow pellets (*n* = 11) (fats 8%, carbohydrates with ash and minerals 67%, proteins 25%; energy 4.70 kcal/g; Labofeed B, Kcynia, Poland) or high-calorie diet (*n* = 46) with nearly three-fold higher content of fats (fats 22%, carbohydrates with ash and minerals 46%, proteins 32%; energy 2.75 kcal/g; Perform, Opti Life, Kronen, Belgium). During the course of the study, animals from both groups had unlimited access to specific diet and drinking water (Ziomber et al., 2009). Daily dietary intake and body weight were recorded throughout the course of the study using standard digital scales. At 24th day of the prestimulation period the rats fed high-calorie diet were randomly divided into four subgroups depending on procedure applied: intact obese rats (without stimulation), obese rats with sham stimulation, obese rats with anodal stimulation of the right prefrontal cortex and obese rats with cathodal stimulation of the left prefrontal cortex. Together with lean intact rats fed standard chow pellets five subgroups were produced. At 25th day of the study the rats designed to either sham or active stimulation underwent epicranial electrode implantation. The rats in surgery received five days for recovery and the first sham or active tDCS was applied at the 30th day of the experiment indicating the beginning of the second part of the study—the stimulation period. During the next seven consecutive days two 10-min sessions of tDCS a day were implemented to each rat from Sh, A, or C group. At 37th day of the study the last stimulation session was employed and all the rats were sacrificed, some 1 h and others 5 h after the last stimulation. Next samples of the brain, blood and other tissues were collected for further examination. Moreover, epididymal fat-pads were excised and weighed with a standard digital scales.

The experiments were conducted in accordance with the National Guide for the Care and Use of Laboratory Animals, and were approved by the Local Ethical Committee on Animal Testing at the Jagiellonian University in Krakow, Poland (approval no. 157/2013). All possible attempts have been made to minimize animal suffering and discomfort, and the number of examined rats was reduced to the necessary minimum.

### tDCS technique

tDCS was carried out with a constant current stimulator (BrainStim, EMS, Bologna, Italy) for continuous application of low currents. The currents were delivered transcranially via an epicranial electrode fixed on the frontal part of the scalp 5 days prior to the tDCS procedure. The electrode was implanted under general anesthesia with 10% ketamine and xylocaine (10 and 3 mg/kg, intramuscularly), using surgical technique described by Liebetanz et al. (2006). A circular electrode (3.5 mm in diameter and 9.6-mm^2^ contact area) with a tubular plastic jacket was placed over the frontal cortex, 3 mm anteriorly from the coronal fissure and 3 mm right or left from the sagittal fissure, fixed with a glass ionomer cement (Ketac Cem, ESPE Dental AG, Seefeld, Germany), and left in place throughout the course of the study. Due to such position of the electrode, electric stimulation could be delivered to the dorsolateral prefrontal cortex (DLPFC). Counter electrode, in form of a conventional rubber plate (10.5 cm^2^, Vermed, Graphic Controls, Poland), was placed on animal's back and stabilized with a corset. We used a unipolar epicranial electrode to prevent bypassing brain currents, unavoidable in the case of two head electrodes placed close to each other. Moreover, the use of asymmetric active electrode enabled us to obtain the highest current density directly underneath. Since the aim of the study was to analyze the effects of repeated tDCS on neurochemical activity of each hemisphere separately, we used an epicranial electrode. Due to its smaller size, electrode of this type can be maintained in place for a longer period of time (Liebetanz et al., 2006; Kamida et al., 2011) in contrast to the latter, its use does not pose a risk of bicephalic stimulation. Therefore, an animal model for tDCS with skin electrode does not fully mimic the procedure used in humans. Prior to the stimulation, the jacket electrode was filled with 0.9% NaCl. Both epicranial electrode and back electrode were connected to the DC stimulator controlled by a dedicated software. During anodal tDCS, current was delivered from epicranial to back electrode, and during cathodal stimulation from back to epicranial electrode. The procedure was conducted without anesthesia. Throughout eight days of stimulation period, each animal was subjected to two 10-min daily sessions of anodal/cathodal stimulation with sham (0 μA) or active (200 μA) electrode. Current intensity was automatically ramped for 10 s to avoid an abrupt switching on/off. During the stimulation, the rats were placed in separate plastic cages and monitored carefully for any behavioral abnormalities.

### Study groups

Six days before tDCS application, the rats on a high calorie-diet, were randomly divided into four subgroups: Ob (*n* = 10)—obese animals, non-exposed to tDCS; Sh (*n* = 11)—obese animals subjected to sham transcranial stimulation; A (*n* = 14)—obese animals subjected to anodal transcranial stimulation of the right prefrontal cortex; and C (*n* = 11)—obese animals subjected to cathodal transcranial stimulation of the left prefrontal cortex. Together with lean animals non-exposed to tDCS (L, *n* = 11), five subgroups were created. The following body weights, just before the first stimulation, were recorded in each group: L - 425 ± 19 g; Ob - 467 ± 31 g; Sh - 464 ± 19 g; A - 466 ± 19 g, and C - 457 ± 21g. The rats received 8 either sham (0 μA twice a day) or active (200 μA twice a day) sessions of tDCS during the stimulation period (from 30th to 37th day of the study). Feeding behavior (body weight gain, epididymal fat pads and food intake) as well as blood metabolic parameters were evaluated from all the study rats while for the brain examinations the study rats were divided into several groups. Separate rats for each time point measurement of monoamine brain metabolism were utilized: 4 rats in each group for measurement 1 h and additional 4 rats in each group–5 h from the last tDCS (*n* = 40). The rest number of rats were designed to the examination of dopamine receptors activity (*n* = 9) and histological verification of tDCS safety (*n* = 8).

The sample size calculations were assisted by the pwr package in R language. For the power level of 0.9, medium group effect (*f* = 0.25) and provided n=5 groups were subjected to ANOVA testing, the total number of 51 rats was proposed. The value obtained was lower than the actual cardinality of the animals used in the experiment.

### Biochemical analysis

Serum levels of leptin and ghrelin were measured using conventional rat ELISA kits (Mouse and Rat Leptin ELISA BioVendor, RayBio Human/Mouse/Rat Ghrelin RayBiotech, respectively). Blood total cholesterol was assessed with the enzymatic method, triglycerides GPO PAP method, and HLD and LDL direct methods on a Roche Cobas c501 analyzer (Roche Diagnostics GmbH, Mannheim, Germany).

### Neurochemical *ex vivo* analysis

For high-performance liquid chromatography (HPLC) examinations, the brain was rapidly removed from the scalp and dissected into different brain structures (left and right site of the frontal cortex and striatum, and additionally nucleus accumbens and hypothalamus) on an ice-cold glass plate. The structures were frozen on solid CO_2_ (−70°C) until used for biochemical assays. Dopamine (DA) and its metabolites, the intraneuronal, 3,4-dihydroxyphenylacetic acid (DOPAC); the extraneuronal, 3-methoxytyramine (3-MT) and final metabolite, homovanillic acid (HVA); and serotonin (5-HT) and its intraneuronal metabolite 5-hydroxyindolacetic acid (5-HIAA) were assayed by means of HPLC with electrochemical detection (ED). The tissue samples were weighted and homogenized in ice-cold 0.1 M trichloroacetic acid containing 0.05 mM ascorbic acid. After centrifugation (10,000 × g, 5 min), the supernatants were filtered through RC58 0.2 μm cellulose membranes (Bioanalytical Systems, West Lafayette, IN, USA). The chromatograph HP 1050 (Hewlett-Packard, Golden, CO, USA) was equipped with Hypersil columns BDS-C18 (4 × 100 mm, 3 μm). The mobile phase consisted of 0.05 M citrate-phosphate buffer, pH 3.5; 0.1 mM EDTA; 1 mM sodium octyl sulfonate and 3.5% methanol. The flow rate was maintained at 1 ml/min. By using autosampler (Ultimate Autosampler 3000), all the specimens were analyzed simultaneously together with checking the standards in every tenth sample. Following each measurement, (in our conditions the time of the distribution of all investigated parameters was about 25 min) additional 10 min period was left wherein the base-line recuperates. DA and 5-HT and their metabolites were quantified by peak area comparisons with standards run on the day of analysis (ChemStation, Hewlett-Packard software computer program) (Antkiewicz-Michaluk et al., 2017).

### Receptor binding studies

#### Dopamine D_1_ receptors

The brain tissues (frontal cortex, striatum and hypothalamus) from individual animals were homogenized in 40 vol of an ice-cold 50 mM Tris-HCl buffer, pH 7.4, using a Polytron disintegrator. The homogenate was centrifuged at 1,000 × g for 15 min, the supernatant was decanted and recentrifuged at 25,000 × g for 30 min, and the resulting pellet was resuspended in the buffer and recentrifuged under the same conditions. The final pellet (fraction P_2_) was used for binding studies. For incubation it was reconstituted in the Tris-HCl buffer pH 7.4, to obtain a final protein concentration of approximately 0.3 mg/ml (Lowry et al., 1951). The radioligand, [^3^H]SCH-23390 ([^3^H](R+)7-chloro-8-hydroxy-3-methyl-1-phenyl-2,3,4,5-tetrahydro-1H-3-benzazepine hydrochloride, NEN, specific activity 85.0 Ci/mmol), was prepared in 5 to 6 concentrations (from 0.05–2.0 nM). The incubation mixture (final volume 600 μl) consisted of 450 μl membrane suspension, 50 μl of [^3^H]SCH-23390 solution, 50 μl 5-HT (1 μM) and 50 μl Tris-HCl buffer without (total binding) or with (unspecific binding) cold SCH-23390 (final concentration 10 μM). All assays were performed in duplicate and incubation proceeded in a shaking water bath, at 30°C for 60 min. The incubation was terminated by rapid filtration through GF/C Whatman fiberglass filters. The filters were than rinsed twice with 5 ml of ice-cold incubation buffer and placed in plastic scintillation minivials. Scintillation fluid (3 ml) was added and the samples were counted for radioactivity in Beckman LS 6500 scintillation counter. Scatchard analysis—the density (Bmax; fmol/mg prot) and affinity (Kd; nM) was calculated by computer analysis program.

#### Dopamine D_2_ receptors

The brain tissues (frontal cortex, striatum and hypothalamus) from individual animals were homogenized in 80 vol of an ice-cold 50 mM Tris-HCl buffer, pH 7.4, using a Polytron disintegrator. The homogenate was centrifuged at 25,000 × g for 20 min, and the resulting pellet was resuspended in the buffer and recentrifuged under the same conditions. The final pellet (fraction P_1_+P_2_) was used for binding studies. For incubation it was reconstituted in the Tris-HCl buffer pH 7.4, and supplemented with 120 mM NaCl, 5 mM KCl, 1 mM CaCl_2_, 1 mM MgCl_2_, 20 μM pargyline and 0.1% ascorbic acid. The membranes are incubated in a shaking water bath at 23°C for 60 min in the presence of the radioligand, [^3^H]Raclopride (PerkinElmer, specific activity 76 Ci/mmol) in 5 concentrations (from 0.05 to 3 nM). The incubation mixture (final volume 550 μl) consisted of 450 μl membrane suspension, 50 μl of [^3^H] Raclopride solution, and 50 μl Tris-HCl buffer without (total binding) or with (unspecific binding) cold Raclopride (final concentration 1 μM). The incubation was terminated by rapid filtration through GF/C Whatman fiberglass filters. The filters were than rinsed twice with 5 ml of ice-cold incubation buffer and placed in plastic scintillation minivials. Scintillation fluid (3 ml) was added and the samples were counted for radioactivity in Beckman LS 6500 scintillation counter. Scatchard analysis (Bmax and Kd) was calculated by computer analysis program.

### Histological processing

After the rats were sacrificed the brain specimens for histopathological examination were obtained, washed in saline, and fixed for 24 h in 8% formalin solution in phosphate buffer (PBS, pH 7.4). Subsequently, the tissue was washed with a running water for 2 h and dehydrated in ascending ethanol gradient (50–100%). Before embedding in paraffin, the specimens were treated with xylene, and then incubated for 2 h at 37°C with xylene and paraffin mixture in 1:1 ratio. Subsequently, the material was treated twice with pure paraffin (2 h at 62°C), embedded in paraffin blocks, cut with a microtome, mounted onto glass slides and dried in an incubator at 37°C. All sections were further processed for hematoxylin–eosin (H&E) staining and histological evaluation was then performed with AXIOPHOT light microscope (Zeiss, Germany).

To minimize the number of animals used in the experiments, only rats sacrificed 5 h after the last stimulation were subjected to histological examinations of the brain.

### Calculations and statistics

Upon ensuring the data obeyed the normal distribution (determined by Shapiro–Wilk test), either parametric or nonparametric tests were used. All the results were presented as mean (±SEM) or median (along with 25–75th percentile). In the case either the behavioral or metabolic data deemed to indicate an agreement with normal distribution and also the data sub-groups were found with equal variances, the parametric one-way analysis of variance (ANOVA), followed by the Tukey *post-hoc* test with the Bonferroni correction, was used. In case of unequal variances the Welch's correction was applied. Not normally distributed data (EFP and HDL levels) were analyzed with nonparametric Kruskal-Wallis test followed by the Dunn *post-hoc* test with the Bonferroni correction. To compare the above parameters at different time points (i.e., before and after stimulation) repeated measures analysis of variance (ANOVA) (with “TIME” as a within-subject factor and “GROUP” as a between-subject factor) followed, when appropriate, by the Tukey *post-hoc* test with the Bonferroni correction was used. To investigate whether the time effect differed between groups, we confirmed the “TIME” × “GROUP” interaction. The Mauchly test of sphericity was performed and the Greenhouse–Geisser correction applied when necessary. As the data for food intake did not fulfill the criterion of normality, the nonparametric alternative of repeated measure ANOVA (mixed models) was used. For doing so, the repeated measure nonparametric ANOVA-type statistic was computed by the in-house procedure implemented in the R language (version 3.4.2) using the nonLD package, whereby the TIME and GROUP effects were assessed while the food intake measurements were repeated over time (Noguchi et al., 2012). Within-group measures of the above parameters were related using either the Pearson's or Spearman's correlation depending on the data distribution (normal or not).

The results of neurochemical studies of the brain (DA, 5-HT and their metabolites) and receptor binding studies were analyzed by one-way analysis of variance (ANOVA) followed, when appropriate, by the Duncan *post-hoc* test. L and Ob groups (intact) were compared with Student T-test. The density (B_max_) and affinity (K_D_) of dopamine D1 and D2 receptors were calculated by nonlinear regression (Scatchard analysis, Statistics Computer Program) (Antkiewicz-Michaluk et al., 2017).

The data were considered statistically significant when *P* < 0.05. Statistical analyses were conducted using IBM Statistics SPSS software (version 24).

The total catabolism rate for dopamine was assessed from the ratio of the final dopamine metabolite concentration, HVA to dopamine concentration and expressed as the catabolic rate index *[HVA]/[DA]x100*; the index of dopamine release as the ratio: *[3-MT]/[DA]*×*100*; and the factor of dopamine re-uptake inhibition as the ratio *[3-MT]/[DOPAC]*×*100*. Analogously, the rate of serotonin metabolism was expressed as the ratio: *[5-HIAA]/[5-HT]*×*100*. The indices were calculated using concentrations from individual tissue samples (Antkiewicz-Michaluk et al., 2017).

## Results

### Behavioral studies

**Figure 1:**
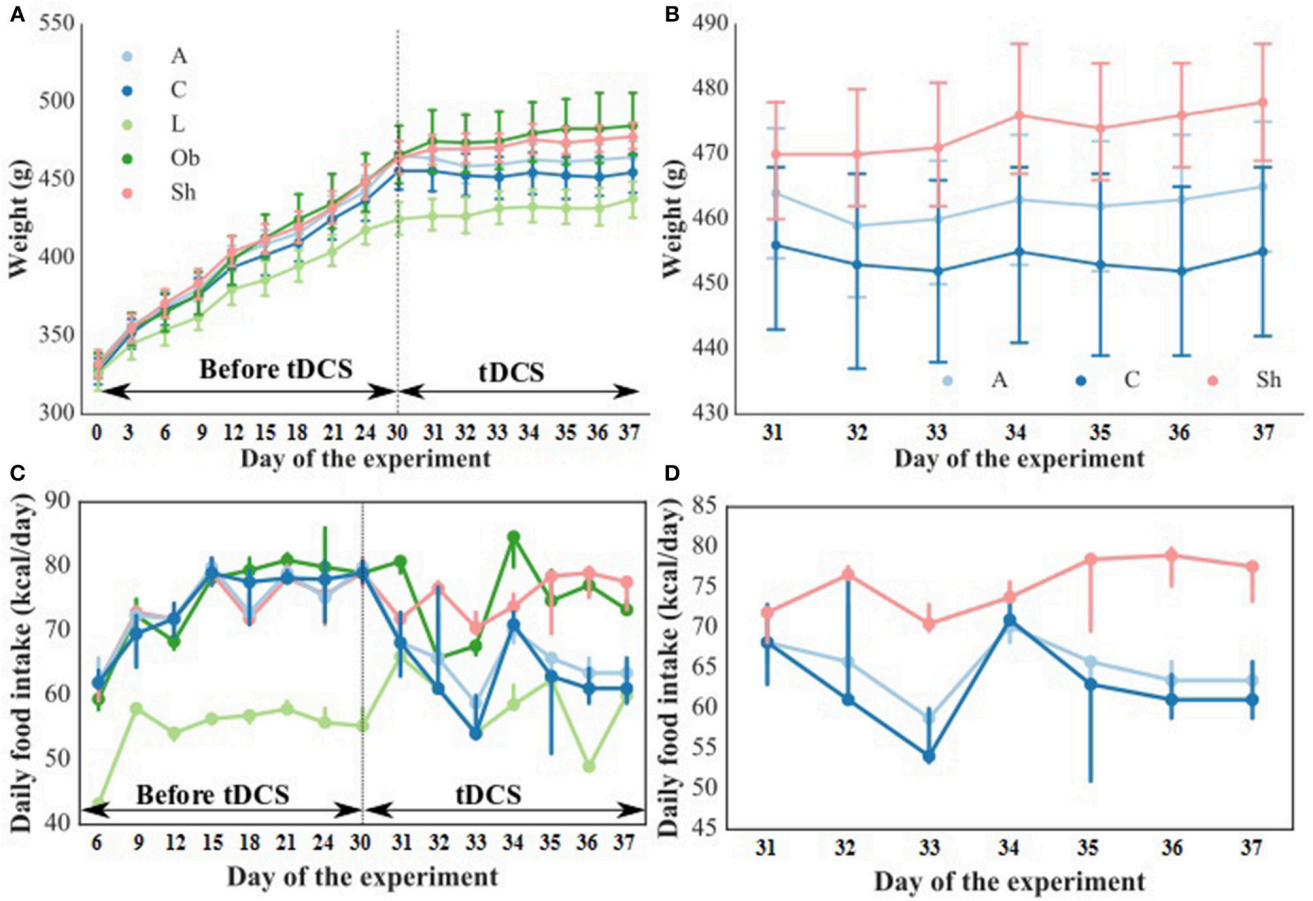
Body weight [g] during the entire experiment **(A)** and during tDCS application **(B)** as well as food intake [kcal/day] during the entire experiment **(C)** and during tDCS application **(D)**. The dashed line indicates the start of tDCS treatment. L, lean intact (*n* = 11); Ob, obese intact (*n* = 10); Sh, obese with sham stimulation (*n* = 11), A, obese with anodal stimulation of the right prefrontal cortex (*n* = 14); C, obese with cathodal stimulation of the left prefrontal cortex (*n* = 11). Data are presented as the mean ± SD and median (25–75th percentile). Repeated measures ANOVA with the Tukey *post-hoc* test (body weight) or repeated measures nonparametric ANOVA (food intake) was used.

**Figure 2:**
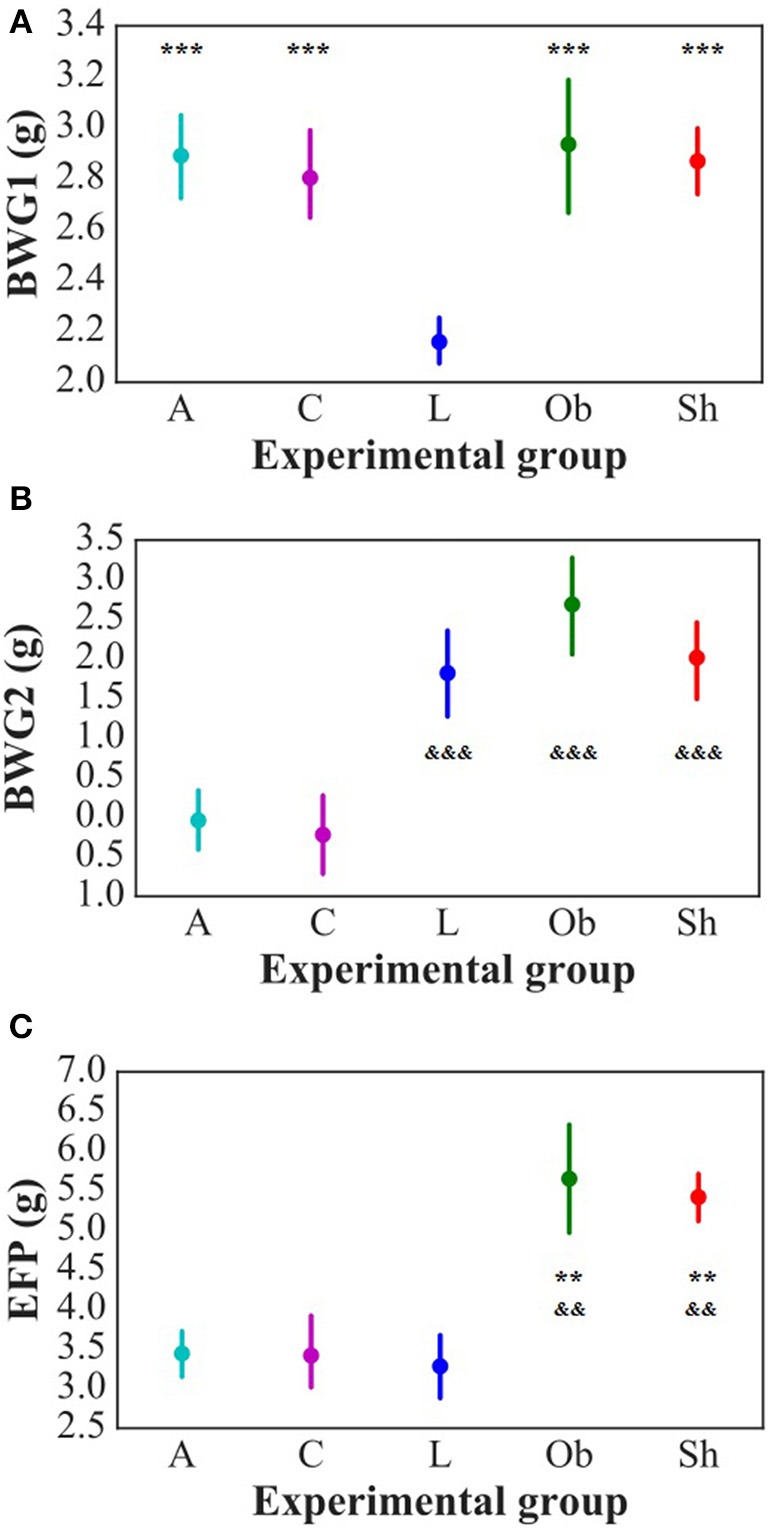
Daily average body weight gain in pre-stimulation period (BWG1) [g/day] **(A)** and during stimulation period (BWG2) [g/day] **(B)** of the study as well as epididymal fat-pads weight (EFP) [g] **(C)**. L, lean intact (*n* = 11); Ob, obese intact (*n* = 10); Sh, obese with sham stimulation (*n* = 11), A, obese with anodal stimulation of the right prefrontal cortex (*n* = 14); C, obese with cathodal stimulation of the left prefrontal cortex (*n* = 11). Data are presented as the mean ± SD. ^*^^*^P < 0.01 vs. L; ^*^^*^^*^P < 0.001 vs. L; ^&&^*P* < 0.01 vs. A and C; ^&*&&*^*P* < 0.001 vs. A and C. The differences between the groups were analyzed with one-way ANOVA followed by the Tukey *post-hoc* test (BWG) or Kruskal-Wallis test followed by the Dunn *post-hoc* test (EFP).

**Figure 3:**
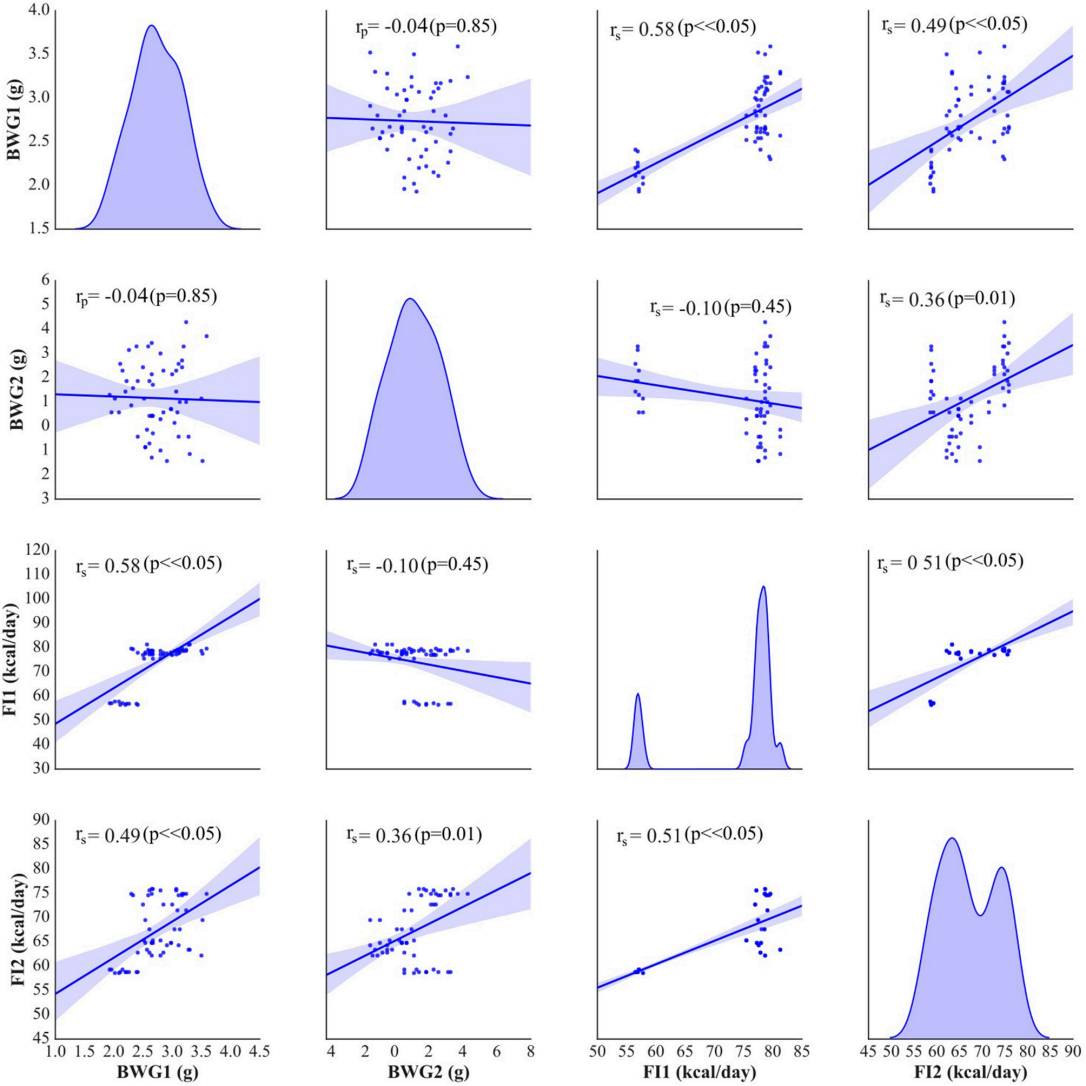
Pearson's or Spearman's correlation between body weight gain (BWG1) [g/day] and the average food intake (FI1) [kcal/day] before stimulation and during tDCS application (BWG2 and FI2, respectively). On the diagonal of the multiple pairwise bivariate distribution plot, the kernel density estimate of the probability density function is shown for each variable subjected to the correlation analysis.

**Figure 4:**
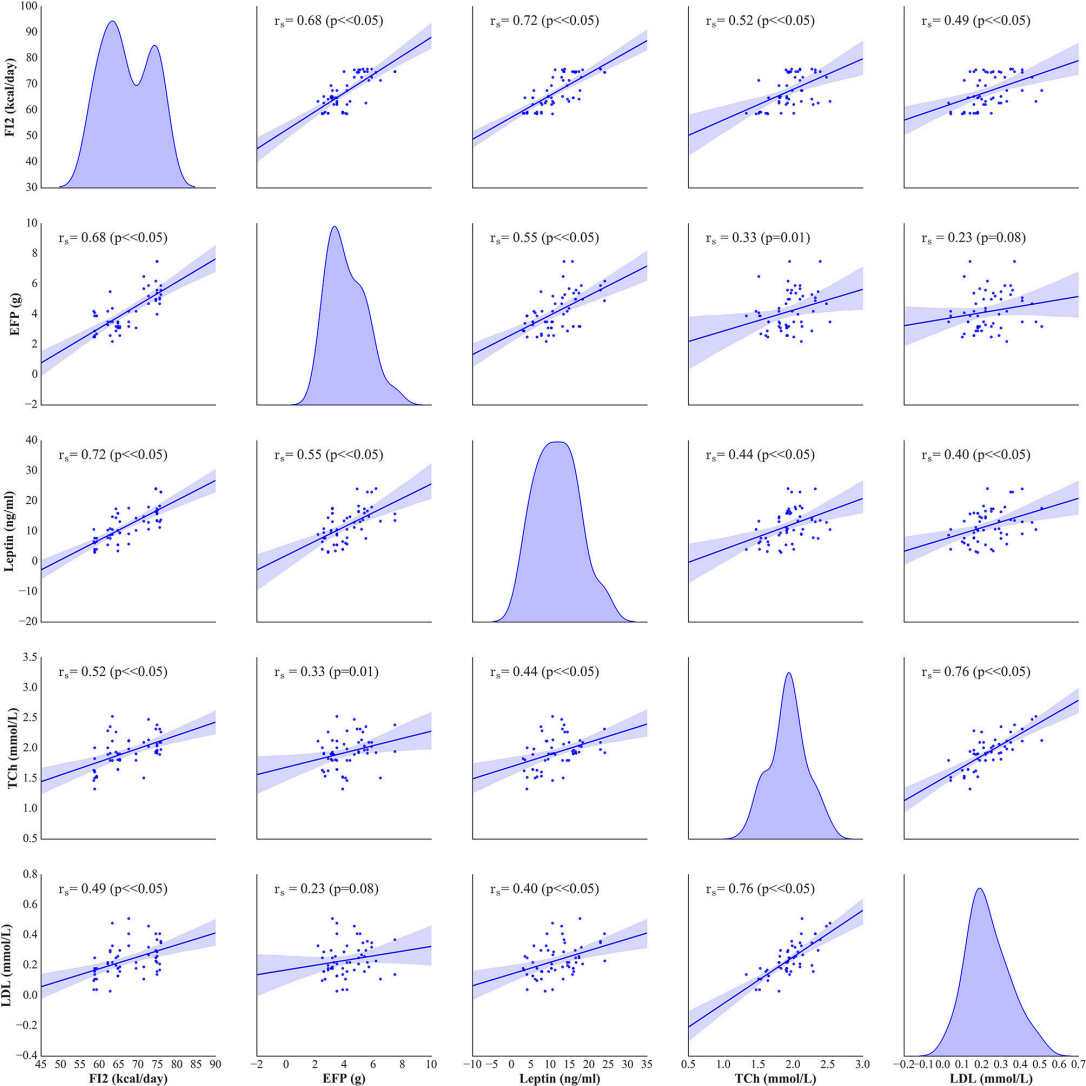
Spearman's correlation between the average food intake (FI2) [kcal/day], epididymal fat-pads (EFP) [g], serum leptin [ng/ml], total cholesterol (TCh) [mmol/L] and LDL [mmol/L] levels. On the diagonal of the multiple pairwise bivariate distribution plot, the kernel density estimate of the probability density function is shown for each variable subjected to the correlation analysis.

The weight and food intake analysis was performed for the pre-stimulation (1–30th day) and post-stimulation periods (31–37th day), separately. Regarding the body weight in the pre-stimulation period, we could find a weak GROUP effect [*F*_(4, 52)_ = 2.8; *p* = 0.034] and a strongly significant TIME effect [*F*_(3, 149)_ = 2,100; *p* < 0.001]. The Ob group had significantly higher body weight (*p* = 0.048), while the rest rats on high-calorie diet (Sh, A, and C) tended (*p* < 0.08) to have increased body weight compared with L rats maintained on standard chow pellets (Figure 1A). The subgroups of rats receiving high-calorie chow (Ob, Sh, A, and C) did not differ significantly in terms of their pre-stimulation body weights. In addition, a significant interaction could be reported between TIME and GROUP [*F*_(11, 149)_ = 5.9; *p* < 0.001]. During the pre-stimulation period, mean daily body weight gain (BWG1) in rats from group L amounted to 2.2 ± 0.1 g, and was significantly lower than in animals kept on high-calorie diet (Figure 2A). As mentioned above, the data for food intake (FI) was analyzed by non-parametric ANOVA-type statistic F'. Regarding pre-stimulation food intake, we could find a strong GROUP effect [*F*'_(2.6)_ = 210; *p* < 0.001] and a strongly significant TIME effect [*F*'_(2.8)_ = 160; *p* < 0.001]. In addition, a significant interaction could be reported between TIME and GROUP [*F*'_(6.6)_ = 11; *p* < 0.001]. It was not a surprise that the rats maintained on a high-calorie diet, despite consuming lower amount of food than those receiving standard chow pellets, had higher daily calorie intake, due to increased energy of the specific diet (Table 1). No statistically significant differences were found in daily food and calorie intakes of rats from subgroups kept on high-calorie diet (Figure 1C; Table 1). Application of active tDCS exerted a significant effect on both body weight gain and food intake in the study rats. A strongly significant effect of TIME [*F*_(2, 129)_ = 32; *p* < 0.001] and GROUP [*F*_(4, 52)_ = 7.1; *p* < 0.001] could be found along with a strong interaction between the TIME and GROUP [*F*_(10, 129)_ = 5.1; *p* < 0.001] in the post-stimulation body weight. The Tukey *post-hoc* test revealed significantly lower body weight of L group compared with Ob, Sh, and A, but not C rats. Animals subjected to anodal tDCS of the right prefrontal cortex or cathodal tDCS of the left prefrontal cortex presented with lesser body weight gains than sham-stimulated or non-stimulated rats (Figures 1B, 2B); however, no significant differences in absolute body weight were found at the end of the study. Regarding the post-stimulation food intake, a strongly significant effect of TIME [*F*'_(2.7)_ = 66; *p* < 0.001] and GROUP [*F*'_(2.7)_ = 260; *p* < 0.001] could be found along with a strong interaction between the TIME and animal GROUP [*F*'_(4.9)_ = 17; *p* < 0.001], as measured by non-parametric ANOVA. During the stimulation period, daily food intake in rats from L, Ob and Sh groups did not differ significantly from its pre-stimulation level (Table 1). In contrast, a significant reduction of food intake was observed in rats from A and C groups. As a result, daily calorie intake in animals from these two groups was not only well below its pre-stimulation level, but turned out to be significantly lower than in sham-stimulated rats (Figures 1C,D). No statistically significant differences in daily food intake were observed between Ob and Sh rats, as well as between A and C rats (Table 1; Figure 1C). We also found positive relationships between body weight gain and the average food intake in both pre-stimulation period of the study and during tDCS application (Figure 3). In addition, the average food intake [kcal/day] during stimulation correlated positively (Spearman's correlation) with epididymal fat pad, leptin, total cholesterol and LDL levels (Figure 4).

**Table 1:** Daily food intake in grams/day and in kcal/day in intact rats on standard diet (L; *n* = 11) and on high-calorie diet (Ob; *n* = 10) and in the rats exposed to sham stimulation (Sh; *n* = 11), anodal stimulation of the right prefrontal cortex (A; *n* = 14) or cathodal stimulation of the left prefrontal cortex (C; *n* = 11) before and during stimulation part of the study.

**Standard/high-calorie diet**	**Before stimulation**		**During stimulation**	
	**[g/day]**	**[kcal/day]**	**[g/day]**	**[kcal/day]**
L	21 (20–21)	57 (55–59)	22 (20–23)	60 (56–62)
Ob	17 (15–17)^**^	78 (70–81)^**^	16 (14–17)^**^^&&^	75 (68–79)^**^^&&^
Sh	16 (15–17)^**^	74 (71–78)^**^	16 (15–17)^**^^&&^	74 (72–78)^**^^&&^
A	16 (15–17)^**^	73 (72–80)^**^	14 (14–15)^**^^##^	66 (64–68)^**^^##^
C	16 (15–17)^**^	76 (71–79)^**^	13 (13–15)^**^^##^	62 (59–71)^##^

*Data are presented as the median (25–75th percentile)*.

** P < 0.01 vs. L;

&& P < 0.01 vs. A and C;

## *P < 0.01 pre- vs. post-stimulation period. Kruskal-Wallis test followed by the Dunn post-hoc test or repeated measures nonparametric ANOVA was used*.

### Metabolic parameters

Administration of high-calorie diet exerted an effect on body adiposity, serum lipids and leptin, but not on ghrelin levels. At the end of the experiment, weight of epididymal fat in non-stimulated animals that remained on high-calorie diet (groups Sh and Ob) was significantly higher than in rats maintained on standard chow pellets (Figure 2C). Furthermore, Sh and Ob rats presented with significantly higher serum concentrations of total cholesterol, LDL and leptin (Table 2; Supplement S1, S2).

**Table 2:** The effects of high-calorie diet (Ob vs. L) and stimulation procedure (Sh vs. A vs. C) on serum total cholesterol [mmol/L], LDL [mmol/L], HDL [mmol/L], triglicerydes (Tg) [mmol/L], serum leptin [ng/mL] and serum ghrelin [ng/mL] concentrations.

	**Total cholesterol [mmol/L]**	**LDL [mmol/L]**	**HDL [mmol/l]**	**Tg [mmol/L]**	**Leptin [ng/mL]**	**Ghrelin [ng/mL]**
L	1.6 ± 0.18	0.13 ± 0.06	0.83 (0.79–0.85)	1.46 ± 0.30	5.8 ± 2.4	39.6 ± 15.3
Ob	2.1 ± 0.17^***^	0.27 ± 0.09^***^	0.88 (0.8–1.55)	1.33 ± 0.50	17.3 ± 3.8^***^^&&^	39.2 ± 12.9
Sh	2.1 ± 0.25^***^	0.29 ± 0.11^***^	1.40 (0.75–1.62)	1.38 ± 0.48	15.7 ± 4.3^***^^&^	39.2 ± 9.7
A	2.0 ± 0.25^***^	0.30 ± 0.13^***^	0.70 (0.61–1.24)	1.27 ± 0.43	10.6 ± 4.9^T^	44.6 ± 8.6
C	2.0 ± 0.23^***^	0.21 ± 0.09	0.78 (0.75–0.88)	1.45 ± 0.38	10.4 ± 3.8^T^	36.3 ± 10.3

*L, lean intact (n = 11); Ob, obese intact (n = 10); Sh, obese with sham stimulation (n = 11), A, obese with anodal stimulation of the right prefrontal cortex (n = 14); C, obese with cathodal stimulation of the left prefrontal cortex (n = 11). Data are presented as the mean ± SD and median (25–75th percentile)*.

T P < 0.1 vs. L;

*** P < 0.001 vs. L;

& P < 0.05 vs. A and C;

&& *P < 0.01 vs. A and C. One-way ANOVA followed by the Tukey post-hoc test or Kruskal-Wallis test followed by the Dunn post-hoc test (HDL) was used*.

**Figure 5:**
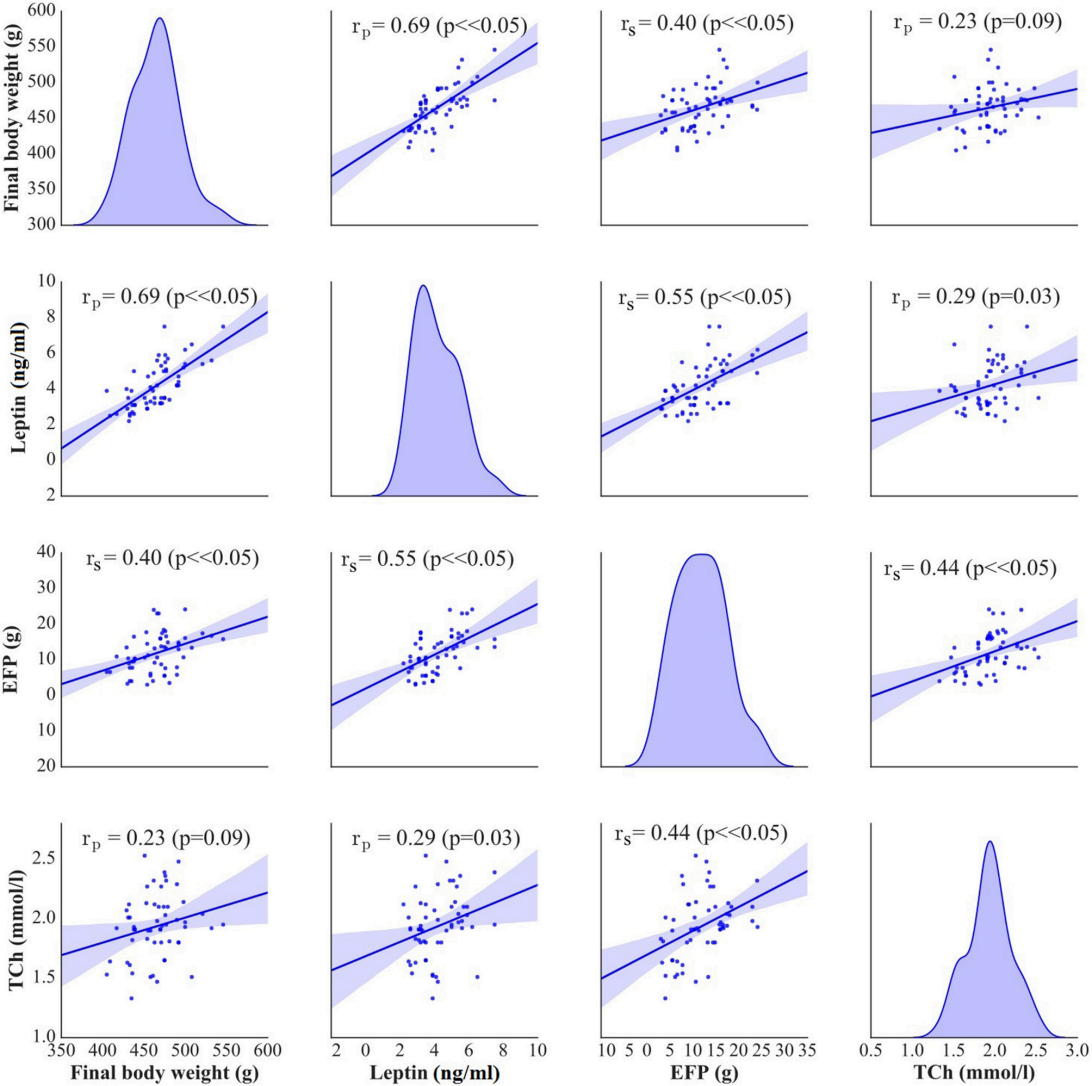
Pearson's or Spearman's correlation between final body weight [g], serum leptin [ng/ml], epididymal fat-pads (EFP) [g], and total cholesterol levels (TCh) [mmol/L]. On the diagonal of the multiple pairwise bivariate distribution plot, the kernel density estimate of the probability density function is shown for each variable subjected to the correlation analysis.

In stimulated rats, a decrease in food intake and lesser body weight gains co-existed with changes in body adiposity, expressed by the weight of epididymal fat. While animals from A and C groups showed a decrease in epididymal fat-pad weight (Figure 2C), no significant changes in this parameter were documented after a sham stimulation; as a result, the weight of epididymal fat in sham-stimulated rats was essentially the same like in non-stimulated group (Ob). Active tDCS did not exert a significant effect on serum lipids; serum concentrations of total cholesterol, LDL, HDL and triglycerides in animals from A and C groups did not differ significantly from those found in other rats maintained on high-calorie diet (Sh and Ob) (Table 2; Supplement S1). In both groups of stimulated rats, a decrease in epididymal fat content co-existed with a reduction of serum leptin level (Table 2; Supplement S2). As a result, mean concentrations of leptin in A and C groups were significantly lower than in sham-stimulated rats; even more pronounced intergroup difference was observed when serum concentrations of leptin in stimulated rats were compared with leptin levels in non-stimulated controls (Ob group). No statistically significant intergroup differences were found in serum concentration of ghrelin (Table 2; Supplement S2). Positive relationships between the final body weight, epididymal fat content, serum leptin, and total cholesterol levels were detected (Figure 5). In addition, correlations between total cholesterol levels, LDL, HDL and triglycerides were found (Supplement S3).

### Neurochemical and receptor binding studies

To analyze the character of tDCS-induced neurochemical changes, we determined concentrations of amine neurotransmitters (dopamine and serotonin), their metabolites, and dopamine receptors, D1 and D2, in various areas of the brain. Then, we used these data to calculate the [metabolite]/[amine] ratios, and the density of dopamine receptors, as a measure of pre- and postsynaptic adaptation.

Non-stimulated rats maintained on standard and high-calorie diets (groups L and Ob, respectively) did not differ significantly in terms of their brain metabolism of monoamines (Table 3; Supplement S4). Irrespective from the time elapsed since the last stimulation, animals subjected to active tDCS (A and C groups) did not differ significantly from sham-stimulated rats in terms of dopamine metabolism rates in all examined brain structures (frontal cortex, dorsal striatum, nucleus accumbens and hypothalamus) (Tables 4–7; Supplement S5–S8). However, 5 h after the last tDCS session, a substantial (25–40%) enhancement of serotonin metabolism was observed in all examined brain structures except hypothalamus of A and C rats when compared with sham-stimulated controls. Repeated anodal tDCS of the right prefrontal cortex contributed to a significant decrease in the density of D2 but not D1 dopamine receptors in the striatum, observed 5 h after the last stimulation (Table 8). Chronic administration of high-calorie diet exerted no effect on the density and affinity of dopamine receptors in examined areas of the brain, as shown by the lack of statistically significant differences in these parameters between L and Sh rats (Table 8).

**Table 3:** Brain concentration of monoamines (DA and 5-HT), their metabolites and the rate of metabolism investigated in FRONTAL CORTEX, DORSAL STRIATUM, and HYPOTHALAMUS in L, lean intact and Ob, obese intact rats.

	**GROUP**		
	***n***	**L**	**Ob**
**FRONTAL CORTEX**			
*DA*	8	729 ± 203	722 ± 134
*DOPAC*	8	198 ± 53	208 ± 37
*HVA*	8	132 ± 35	138 ± 19
*3-MT*	8	35 ± 11	30 ± 6
*[HVA]/[DA]*	8	18 ± 3	19 ± 3
*[3-MT]/[DA]*	8	5 ± 1.2	4 ± 1.0
*5-HT*	8	268 ± 30	294 ± 53
*5-HIAA*	8	148 ± 31	164 ± 39
*[5-HIAA]/[5-HT]*	8	55 ± 9	56 ± 8
**DORSAL STRIATUM**			
*DA*	8	13, 153 ± 1, 300	13, 614 ± 1, 776
*DOPAC*	8	1, 984 ± 254	2, 094 ± 360
*HVA*	8	1, 159 ± 236	1, 176 ± 238
*3-MT*	8	507 ± 133	495 ± 118
*[HVA]/[DA]*	8	9 ± 1.5	8 ± 1
*[3-MT]/[DA]*	8	4 ± 1	4 ± 0.8
*5-HT*	8	325 ± 205	216 ± 47
*5-HIAA*	8	171 ± 40	182 ± 46
*[5-HIAA]/[5-HT]*	8	68 ± 28	84 ± 10
**HYPOTHALAMUS**			
*DA*	8	459 ± 66	463 ± 76
*DOPAC*	8	116 ± 13	124 ± 21
*HVA*	8	11 ± 2	10 ± 1
*3-MT*	8	16 ± 1	13 ± 3
*[HVA]/[DA]*	8	2 ± 0.3	2 ± 0.4
*[3-MT]/[DA]*	8	3 ± 0.6	3 ± 0.5
*5-HT*	8	258 ± 17	261 ± 15
*5-HIAA*	8	170 ± 12	168 ± 10
*[5-HIAA]/[5-HT]*	8	66 ± 4	64 ± 3

*Data are presented as the mean ± SD; (n) – the number of animals per group. Student T-test was used. No differences were detected between the examined groups (P > 0.05)*.

**Table 4:** The effect of repetitive anodal (A) and cathodal (C) tDCS on the brain concentration of monoamines (DA and 5-HT), their metabolites and on the rate of metabolism at different time point after the last stimulation investigated in the right and left FRONTAL CORTEX.

**AMINES AND METABOLITES (ng/g tissue)**		***FRONTAL CORTEX*** **(1 h after the last tDCS)**					
	***n***	**Sh**		**A**		**C**	
		**Right side**	**Left side**	**Right side**	**Left side**	**Right side**	**Left side**
*DA*	4	629 ± 80	601 ± 57	558 ± 99	456 ± 87	642 ± 56	701 ± 39
*DOPAC*	4	200 ± 23	201 ± 20	162 ± 36	157 ± 25	190 ± 8	210 ± 10
*HVA*	4	127 ± 13	133 ± 13	120 ± 14	114 ± 14	136 ± 2	162 ± 3
*3-MT*	4	26 ± 2	23 ± 2	20 ± 5	16 ± 3	28 ± 5	25 ± 3
*[HVA]/[DA]*	4	21 ± 2	22 ± 1	23 ± 2	25 ± 3	22 ± 2	23 ± 1
*[3-MT]/[DA]*	4	4 ± 0.5	4 ± 0.3	4 ± 0.6	3 ± 0.3	4 ± 0.6	3 ± 0.3
*5-HT*	4	248 ± 26	270 ± 18	424 ± 109	237 ± 25	309 ± 12	330 ± 11
*5-HIAA*	4	151 ± 10	154 ± 10	175 ± 17	140 ± 17	179 ± 7	193 ± 6
*[5-HIAA]/[5-HT]*	4	62 ± 2	57 ± 1	51 ± 11	59 ± 4	58 ± 1	58 ± 0.7
***FRONTAL CORTEX*** **(5 h after the last tDCS)**							
*DA*	4	667 ± 99	690 ± 75	907 ± 92	550 ± 61	920 ± 116	807 ± 26
*DOPAC*	4	177 ± 20	183 ± 10	231 ± 19	185 ± 33	289 ± 68	276 ± 54
*HVA*	4	115 ± 8	112 ± 5	135 ± 12	115 ± 21	166 ± 38	160 ± 27
*3-MT*	4	44 ± 6	41 ± 5	55 ± 6	44 ± 12	63 ± 23	64 ± 19
*[HVA]/[DA]*	4	18 ± 2	17 ± 2	15 ± 1	21 ± 4	15 ± 2	20 ± 3
*[3-MT]/[DA]*	4	7 ± 0.3	6 ± 0.1	6 ± 0.3	8 ± 1.5	7 ± 0.6	8 ± 1.8
*5-HT*	4	291 ± 40	284 ± 7	227 ± 30	226 ± 19	202 ± 8	208 ± 31
*5-HIAA*	4	149 ± 17	155 ± 9	154 ± 14	140 ± 4	153 ± 8	151 ± 21
*[5-HIAA]/[5-HT]*	4	52 ± 3	54 ± 2	69 ± 3^**^	63 ± 4^*^	76 ± 4^**^	74 ± 5^*^

*Data are presented as the mean ± SD; (n) – the number of animals per group. One-way analysis of variance (ANOVA) with the Duncan post-hoc test was used*.

* P < 0.05 vs sham stimulation (Sh);

** *P < 0.01 vs Sh*.

**Table 5:** The effect of repetitive anodal (A) and cathodal (C) tDCS on the concentration of monoamines (DA, and 5-HT), their metabolites and on the rate of metabolism at different time point after the last stimulation investigated in the right and left DORSAL STRIATUM.

**AMINES AND METABOLITES (ng/g tissue)**		***DORSAL STRIATUM*** **(1 h after the last tDCS)**					
	***n***	**Sh**		**A**		**C**	
		**Right side**	**Left side**	**Right side**	**Left side**	**Right side**	**Left side**
*DA*	4	13, 453 ± 616	11, 903 ± 583	13, 323 ± 949	11, 956 ± 825	15, 260 ± 848	13, 474 ± 853
*DOPAC*	4	2, 258 ± 128	1, 832 ± 80	1, 940 ± 221	1, 890 ± 171	2, 424 ± 87	2, 091 ± 221
*HVA*	4	1, 362 ± 118	964 ± 69	1, 351 ± 97	938 ± 112	1, 569 ± 101	1, 259 ± 78
*3-MT*	4	526 ± 29	429 ± 32	424 ± 37	309 ± 62	473 ± 5	439 ± 26
*[HVA]/[DA]*	4	9 ± 0.5	8 ± 0.5	10 ± 0.4	9 ± 0.5	10 ± 0.3	9 ± 0.4
*[3-MT]/[DA]*	4	3 ± 0.1	4 ± 0.2	3 ± 0.1	3 ± 0.4	3 ± 0.1	3 ± 0.2
*5-HT*	4	233 ± 11	212 ± 22	199 ± 24	198 ± 19	216 ± 11	243 ± 11
*5-HIAA*	4	212 ± 4	200 ± 24	174 ± 23	171 ± 16	190 ± 14	227 ± 15
*[5-HIAA]/[5-HT]*	4	92 ± 5	94 ± 2	87 ± 3	91 ± 2	88 ± 3	93 ± 2
***DORSAL STRIATUM*** **(5 h after the last tDCS)**							
*DA*	4	13, 502 ± 269	12, 850 ± 410	12, 349 ± 653	13, 501 ± 966	14, 828 ± 361	12, 415 ± 988
*DOPAC*	4	1, 851 ± 93	1, 713 ± 34	1, 785 ± 180	2, 068 ± 86	2, 215 ± 67	1, 893 ± 218
*HVA*	4	1, 108 ± 60	937 ± 55	944 ± 35	930 ± 67	1, 166 ± 76	968 ± 113
*3-MT*	4	632 ± 32	617 ± 19	592 ± 41	677 ± 80	711 ± 30	624 ± 64
*[HVA]/[DA]*	4	8 ± 0.3	7 ± 0.6	8 ± 0.1	7 ± 0.2	8 ± 0.4	8 ± 0.5
*[3-MT]/[DA]*	4	5 ± 0.2	5 ± 0.1	5 ± 0.2	5 ± 0.2	5 ± 0.3	5 ± 0.4
*5-HT*	4	238 ± 15	240 ± 22	224 ± 26	230 ± 28	238 ± 23	215 ± 17
*5-HIAA*	4	193 ± 15	205 ± 13	209 ± 22	227 ± 18	233 ± 22	227 ± 16
*[5-HIAA]/[5-HT]*	4	81 ± 2	85 ± 2	94 ± 5^*^	100 ± 5^*^	98 ± 2^**^	106 ± 5^**^

*Data are presented as the mean ± SD; (n) – the number of animals per group. One-way analysis of variance (ANOVA) with the Duncan post-hoc test was used*.

* P < 0.05 vs. sham stimulation (Sh);

** *P < 0.01 vs. Sh*.

**Table 6:** The effect of repetitive anodal (A) and cathodal (C) tDCS on the concentration of monoamines (DA, and 5-HT), their metabolites and on the rate of metabolism at different time point after the last stimulation investigated in NUCLEUS ACCUMBENS.

**AMINES AND METABOLITES (ng/g tissue)**		***NUCLEUS ACCUMBENS*** **(1 h after the last tDCS)**		
	**n**	**Sh**	**A**	**C**
*DA*	4	10, 074 ± 595	8, 995 ± 281	9, 061 ± 509
*DOPAC*	4	2, 221 ± 170	2, 148 ± 57	2, 084 ± 264
*HVA*	4	914 ± 123	931 ± 93	1, 025 ± 95
*3-MT*	4	298 ± 19	250 ± 23	262 ± 19
*[HVA]/[DA]*	4	9 ± 0.5	10 ± 0.7	11 ± 0.6
*[3-MT]/[DA]*	4	3 ± 0.2	3 ± 0.2	3 ± 0.2
*5-HT*	4	269 ± 46	331 ± 97	253 ± 20
*5-HIAA*	4	96 ± 16	117 ± 8	90 ± 10
*[5-HIAA]/[5-HT]*	4	38 ± 3	36 ± 9	36 ± 4
***NUCLEUS ACCUMBENS*** **(5 h after the last tDCS)**				
*DA*	4	9, 137 ± 561	7, 594 ± 541	8, 897 ± 855
*DOPAC*	4	1, 657 ± 109	1, 790 ± 306	1, 954 ± 61
*HVA*	4	723 ± 32	606 ± 63	754 ± 48
*3-MT*	4	422 ± 24	355 ± 28	429 ± 25
*[HVA]/[DA]*	4	8 ± 0.6	8 ± 0.4	8 ± 0.6
*[3-MT]/[DA]*	4	5 ± 0.4	5 ± 0.2	5 ± 0.4
*5-HT*	4	199 ± 17	131 ± 27	134 ± 26
*5-HIAA*	4	82 ± 7	76 ± 12	78 ± 6
*[5-HIAA]/[5-HT]*	4	41 ± 1	60 ± 3^*^	59 ± 7^*^

*Data are presented as the mean ± SD; (n) – the number of animals per group. One-way analysis of variance (ANOVA) with the Duncan post-hoc test was used*.

* *P < 0.05 vs. sham stimulation (Sh)*.

**Table 7:** The effect of repetitive anodal (A) and cathodal (C) tDCS on the concentration of monoamines (DA, and 5-HT), their metabolites and on the rate of metabolism at different time point after the last stimulation investigated in HYPOTHALAMUS.

**AMINES AND METABOLITES (ng/g tissue)**		***HYPOTHALAMUS*** **(1 h after the last tDCS)**		
	**n**	**Sh**	**A**	**C**
*DA*	4	436 ± 63	434 ± 32	453 ± 73
*DOPAC*	4	122 ± 16	108 ± 8	120 ± 27
*HVA*	4	9 ± 0.7	10 ± 0.8	6 ± 1
*3-MT*	4	15 ± 5	16 ± 2	13 ± 3
*[HVA]/[DA]*	4	2 ± 0.3	2 ± 0.3	2 ± 0.4
*[3-MT]/[DA]*	4	3 ± 0.6	4 ± 0.6	3 ± 0.2
*5-HT*	4	268 ± 18	294 ± 17	294 ± 10
*5-HIAA*	4	167 ± 10	169 ± 12	180 ± 10
*[5-HIAA]/[5-HT]*	4	63 ± 0.8	57 ± 3	61 ± 2
***HYPOTHALAMUS*** **(5 h after the last tDCS)**				
*DA*	4	385 ± 41	338 ± 20	364 ± 32
*DOPAC*	4	95 ± 13	96 ± 12	104 ± 5
*HVA*	4	11 ± 0.6	8 ± 0.5	9 ± 1
*3-MT*	4	14 ± 3	11 ± 2	15 ± 5
*[HVA]/[DA]*	4	3 ± 0.4	2 ± 0.1	2 ± 0.4
*[3-MT]/[DA]*	4	4 ± 1	3 ± 1	4 ± 1
*5-HT*	4	255 ± 15	249 ± 43	259 ± 5
*5-HIAA*	4	163 ± 12	172 ± 18	181 ± 6
*[5-HIAA]/[5-HT]*	4	65 ± 2	73 ± 9	70 ± 2

*Data are presented as the mean ± SD; (n) – the number of animals per group. One-way analysis of variance (ANOVA) with, if necessary, the Duncan post-hoc test was used. No differences between tested groups were detected*.

**Table 8:** The effect of repetitive anodal tDCS (A) of the right prefrontal cortex on the density B_max_ and affinity K_D_ of dopamine D_1_ and D_2_ receptors IN THE FRONTAL CORTEX and DORSAL STRIATUM of rat.

**Receptor subclass**	**Group**	***FRONTAL CORTEX***		***DORSAL STRIATUM***	
		**B_max_ (fmol/mg prot)**	**K_D_ (nM)**	**B_max_ (fmol/mg prot)**	**K_D_ (nM)**
	L	157 ± 18	0.6 ± 0.09	650 ± 52	0.3 ± 0.05
DA D_1_	Sh	163 ± 19	0.6 ± 0.08	650 ± 54	0.4 ± 0.04
*[^3^H]SCH_23390_*	A 5h	148 ± 11	0.5 ± 0.07	568 ± 28	0.4 ± 0.01
*F*_(3/8)_		*0.637 ns*	*1.133 ns*	*0.871 ns*	*1.070 ns*
	L	23 ± 5	2.5 ± 0.5	75 ± 6	0.9 ± 0.06
DA D_2_	Sh	19 ± 5	2.0 ± 0.8	68 ± 5	0.9 ± 0.02
*[3H]Raclopride*	A 5h	19 ± 6	2.0 ± 1.1	57 ± 9^*^^&^	0.8 ± 0.05
*F*_(3/8)_		*0.201 ns*	*0.870 ns*	***4.817 P** < **0.03***	*1.247 ns*

*L, lean intact; Sh, obese with sham stimulation; A5h, obese with anodal stimulation of the right prefrontal cortex; the rats were sacrificed 5 h after the last stimulation. Data are presented as the means ± SD. B_max_ (fmol/mg protein) and K_D_ (nmol/liter) were calculated by nonlinear regression (Scatchard analysis, Statistics Computer Program)*.

* P < 0.05 vs L;

& *P < 0.05 vs Sh. One-way analysis of variance (ANOVA) with the Duncan post-hoc test was used. Neither differences in the density nor affinity of dopamine D1 (DAD1) and D2 (DAD2) receptors in FRONTAL CORTEX between the tested groups were detected (GROUP effect DAD1: F_(3, 8)_ = 0.637, ns or F_(3, 8)_ = 1.133, ns for B_max_ or K_D_, respectively and GROUP effect DAD2: F_(3, 8)_ = 0.201, ns or F_(3, 8)_ = 0.870, ns for B_max_ or K_D_, respectively); ns, p > 0.05. The rats with anodal tDCS (A5h) had significantly decreased density, but not affinity of DAD2 in DORSAL STRIATUM compared with both L and Sh rats [GROUP effect: F_(3, 8)_ = 4.817, p < 0.03]*.

### Histological processing

Histological examination of the brain tissue was performed in eight rats: A (*n* = 3), C (*n* = 3) and Ob (*n* = 2) 5 h after the last stimulation. The brain tissue was examined to detect possible injury following active tDCS application. Macroscopically, no abnormalities were observed. No signs of neurotrauma, edema, hematoma of other pathological changes were detected by light microscopy in rats after anodal tDCS of the right prefrontal cortex or cathodal tDCS of the left prefrontal cortex (Supplement S9).

## Discussion

To the best of our knowledge, this is the first study to analyze the effects of repeated tDCS on behavioral, metabolic and neurochemical parameters in animals maintained chronically on high-calorie diet. In this study, both anodal tDCS of the right prefrontal cortex and cathodal tDCS of the left prefrontal cortex showed similar effectiveness in reduction of food intake and prevention of body weight gain in rats kept on high-calorie diet. The tDCS-induced changes in feeding behavior co-existed with a decrease in body adiposity (weight of epididymal fat-pads) and serum leptin levels. The second important finding of our study is the observation that exposure to active tDCS resulted in enhancement of serotonin system in reward related-brain areas, such as frontal cortex, dorsal striatum and nucleus accumbens, and contributed to a decrease in dopamine receptor D_2_ density in the dorsal striatum, observed 5 h after the last stimulation. This implies that these are changes in cerebral metabolism of serotonin and dopamine, which may play a role in etiopathogenesis of obesity and at least partially explain the mechanisms through which tDCS interferes with energy homeostasis.

There is an increasing interest in tDCS application for modifying appetite in patients with obesity. Unfortunately, little is still know about its effects either at the behavioral or biochemical levels in individuals with obesity. Therefore we aimed in our study to determine the action of tDCS in obese animals which is now tremendously important for allowing this novel therapy to be used optimally in a clinical setting. Thus tDCS in our experiment was exclusively employ to the rats on high-calorie diet and the data indicate an interaction of tDCS and hypercalorie diet.

The choice of stimulation protocol was justified by the paucity of data on the influence of repeated tDCS on energy homeostasis in obese subjects. Most previous studies analyzed the effects of single tDCS session on food craving in normal-weight (Fregni et al., 2008; Lapenta et al., 2014) or overweight/obese subjects (Goldman et al., 2011; Montenegro et al., 2012; Kekic et al., 2014). The studies involving repetitive tDCS, if any, analyzed the effects of the stimulation on food craving or appetite in lean animals (Macedo et al., 2016) and humans (Jauch-Chara et al., 2014). Published evidence suggests that parameters of stimulation used in this study (current intensity, duration and number of stimulation cycles, diameters of active and counter electrodes) were safe and non-harmful (Liebetanz et al., 2009; Spezia Adachi et al., 2012). In a previous study of rats, cathodal stimulation with current density greater than 142.9 A/m^2^ posed a risk of brain lesions (Liebetanz et al., 2009), and authors of a more recent experiment (Jackson et al., 2017) reported a brain damage after anodal tDCS with 20.0 A/m^2^ current density. Although current density used in our experiment (20.8 A/m^2^) slightly exceeded the latter value, brain tissue specimens from the study rats showed no macroscopic or microscopic evidence of abnormalities. Moreover, animals subjected to the stimulation showed no behavioral anomalies or signs of discomfort. The lack of harmful effects in our study rats can be partially explained by markedly shorter stimulation time; in our experiment, a single stimulation session lasted no longer than 10 min, as compared to up to 60-min anodal tDCS in the previously mentioned study conducted by Jackson et al. Our result further emphasize that the lesion area is not simply predicted by electrode current density and so not by charge density. Location of the electrodes and character of the stimulation were chosen based on previously published observations that both anodal and cathodal tDCS may influence appetite and food craving (Fregni et al., 2008; Lapenta et al., 2014). Moreover, in line with one theory (Alonso-Alonso and Pascual-Leone, 2007) hyperphagia and obesity result from relative hypoactivity of the right hemisphere. To verify this hypothesis, we either stimulated right prefrontal cortex with anodal tDCS or inhibited left prefrontal cortex with cathodal tDCS in obese animals.

Exposure to fat-rich diet resulted in development of typical metabolic abnormalities (Olsen et al., 2017). Energy intake and body weight gain in rats maintained on high-calorie diet were greater than in animals fed with a standard chow. This resulted in a significant increase in total body weight and body adiposity expressed by the weight of epididymal fat-pads. Moreover, high-fat diet contributed to an increase in serum concentrations of total cholesterol, LDL and leptin. Positive correlation between the weight of epididymal fat-pads and leptin concentration implies that the increase in the latter parameter was proportional to the degree of body adiposity.

Anodal tDCS of the right prefrontal cortex and cathodal tDCS of the left prefrontal area exerted similar effects on feeding behavior, metabolic parameters and brain activity of monoamine neurotransmitters in rats maintained on high-calorie diet. This implies that interhemispheric interactions and complex cortical-subcortical connectivity are key determinants of appetite control. In a previous study (Wang et al., 2006) implantable gastric stimulator (IGS), a system which generates electric signals to induce satiety in obese subjects, was shown to enhance right hemispheric activity, especially in the prefrontal cortex and hippocampus. Some evidence suggests that also left dorsolateral prefrontal cortex (DLPFC) may play a role in the control of craving (Hayashi et al., 2013), and right DLPFC seems to be involved in the inhibitory control of emotional impulses (Pripfl et al., 2013). However, it is unclear which region of the brain was primarily affected by tDCS in our study rats. Repeated stimulation, both anodal right tDCS and cathodal left tDCS, contributed to reduction of food intake, lesser body weight gain, and a decrease in body adiposity and serum leptin levels. These findings are consistent with the results of previous human (Montenegro et al., 2012; Jauch-Chara et al., 2014; Kekic et al., 2014; Lapenta et al., 2014) and animal (Macedo et al., 2016) studies, in which a single or repeated tDCS exerted a significant effect on craving and appetite. Recently, Ray et al. (2017) demonstrated for the first time that repeated tDCS contributed to a decrease in food craving and appetite in adults with trunk obesity. Interestingly, changes in feeding behavior, body adiposity and leptin levels in our study rats were accompanied by an increase in the activity of brain serotonin system. The concentrations of serotonin and its metabolite, 5-HIAA were not significantly changed after tDCS instead its metabolism was essentially increased 5 h after tDCS in the all investigated brain structures, except the hypothalamus. Such intensification of serotonin metabolism marked as index [5-HIAA]/[5-HT] is closely connected with serotonin release to the extracellular area and activation of serotonin system in the investigated brain structures. Our data presented the real stimulant effect of tDCS on serotonin system and are in agreement with others showing the important role of serotonin in appetite control as an inhibitor of calorie intake in rodents (López-Alonso et al., 2007; Lam et al., 2008). Enhanced metabolism of serotonin (an increase in 5-HIAA/5-HT ratio) in the frontal cortex (FCx), dorsal striatum (STR) and nucleus accumbens (NAc) was demonstrated at 5 h, but not 1 h after the last stimulation. Basing on our results one can suspect that tDCS effects on the brain metabolism are present with a delay. It is challenging to speculate what is the responsible mechanism. However, it is plausible that tDCS applied with protocol like in our study could indirectly affect the monoamine metabolism, and additional time was required to exert the effects in the examined brain structures. This hypothesis is in agreement with in vivo microdialysis animal studies (Keck et al., 2002; Tanaka et al., 2013) which showed the earliest significant increase in brain monoamine releasing 100 or 150 min after tDCS or repetitive transcranial magnetic stimulation (rTMS). However it should be underlined that these invasive procedures, which directly measure biogenic amines via a probe inserted into a target brain region after one session of the brain stimulation substantially differ from our study protocol of chronic tDCS application. Surprisingly, activation of serotonin system was observed in both right and left hemisphere, after either cathodal or anodal tDCS. This confirms presence of interhemispheric connectivity and implies that the effects of tDCS are not limited solely to cortical areas located directly under the electrode, but due to presence of cortico-subcortical interactions may also extend onto distant areas of the brain (Lapenta et al., 2014). The role of serotonin in appetite control is well established. 5-HT drugs were shown to reduce calorie intake in rodents (Lam et al., 2008) and humans (Smith et al., 2014), promoting satiety and decreasing hunger. The recent investigation into the central serotonergic regulation of energy balance pointed both hypothalamic and non-hypothalamic i.e., reward brain structures that have been implicated in serotonin-mediated feeding behavior control (Donovan and Tecott, 2013). Although hypothalamus is recognized as a key region for serotonin action, we did find significant changes in hypothalamic activity of 5-HT neither 1 h nor 5 h after application of the last session of tDCS. It seems that tDCS exerted more potent changes in reward-related pathways rather than hypothalamus-dependent homeostatic control of metabolism in our rats. However, these results do not exclude the possibility that hypothalamic serotoninergic system response may occur later i.e., 24 h after the last stimulation. Furthermore, Farr et al. (2016) did not observe any changes in hypothalamic activity after administration of lorcaserin, an agonist of serotonin 5-hydroxytryptamine 2c receptor, being effective in obesity treatment. In this study, we did not observe significant changes within dopaminergic system of rats at any time point after anodal or cathodal stimulation except a decrease in D2 receptor density in dorsal STR detected 5 h after the last anodal stimulation. The involvment of dopamine in feeding control is thought to be well established (Volkow et al., 2011). It is a key neurotransmitter modulating rewarding effects of food associated with “wanting” rather than “liking” of food. Ingestion of palatable food has been shown to release DA in the dorsal striatum (Small et al., 2003) and enhanced dopaminergic neurotransmission is proposed to be one of the driving forces underlying obsessive eating behavior (Garfield and Heisler, 2009). On the other hand, under-activation of dopaminergic tracts by the palatability food intake might lead to consecutive overconsumption to compensate for the weak DA inputs. Indeed, downregulation of striatal D2 receptors has been already reported in overweight humans (Wang et al., 2001) and rodents (Huang et al., 2006), and interpreted as a neuroadaptive response to overconsumption of palatable foods. Therefore, a decrease in dopamine D2 receptor density in dorsal STR observed 5 h after the last anodal stimulation in our study may have implications to overweight rats. However, despite the formulation of reward deficit theory of obesity (Val-Laillet et al., 2015), the role of D2 receptors in this condition is yet to be established. Available evidence in this matter is inconclusive: while some studies demonstrated that lower DA D2/3R binding in caudate and amygdala correlated with an increase in BMI (Savage et al., 2014) other experiments showed an opposite relationship (Dunn et al., 2012) or found no association between striatal DA D2 binding and BMI (Eisenstein et al., 2013). Furthermore, no difference was reported in DA D2/D3R availability in obese and non-obese women (Karlsson et al., 2015). Since both serotoninergic and dopaminergic systems show the interaction with other controls of food intake, the involvement of other neurotransmitters and systems (i.e., glutamate and GABAergic system, cholinergic system, opioids, cannabinoid system) in mediation of feeding behavior cannot be excluded in our rat study.

## Conclusion

Our study demonstrated that tDCS is a non-invasive and effective technique to modulate feeding behavior in rats maintained on high-calorie diet. Either anodal tDCS of the right DLPFC or cathodal tDCS of the left DLPFC turned out to be equally effective in reduction of appetite and body adiposity, as well as in control of other metabolic abnormalities typical of obesity. Post-tDCS changes in feeding behavior co-existed with upregulation of serotonin activity in examined structures of the brain, as well as with a significant decrease in the density of dopamine receptors D2 in the dorsal striatum. We can conclude that it is alternation of serotoninergic and dopaminergic system activity, which has implications for feeding control and may at least partially explain the mechanism being involved in tDCS-mediated regulation of feeding behavior. Despite the fact we did not show any changes in brain DA metabolism activity, our results do not exclude the possibility of the delayed response of dopamine system to repetitive tDCS application and its contribution to metabolic effects. Further research is needed to verify whether the same relationships exist in humans.

## Study limitations and perspectives

Although this study provided an important data about behavioral, metabolic and neurochemical mechanisms activated in response to tDCS, it had also several potential limitations. All experiments were carried out solely in male rats; therefore, future studies should verify if the hereby presented findings can be extrapolated to female rats. Although, contrary to human studies, in which obesity tends to be more prevalent in females, some of the animal studies showed that female rats gain their body weights slowly and are not convenient model of obesity. To reduce the number of examined rats to the necessary minimum (3R), tDCS was employed exclusively to obese rats and we did not explore the effects of tDCS in lean animals. Another important issue is that caution should be preserved while extrapolating the results to humans. This is due to the fact that although we applied current of lower intensity than typically used clinical studies (0.2 vs. 2.0 mA, respectively), due to small size of the electrode, current density, although not harmful, was substantially greater than in a clinical setting (20.8 vs. 0.57 A/m^2^, respectively). These discrepancies might have some important implications, resulting in different areas being stimulated in rats than in humans. Importantly, in this study we did not analyze long-term effects of tDCS on feeding behavior, metabolic parameters and on neurobiochemical properties of the brain; therefore, future studies should center around the identification of both minimal and the most effective currents, as well as around potential long-term consequences of tDCS in higher number of animals. Finally, aside from dopamine and serotonin, the effects of tDCS on feeding behavior are likely mediated by other substances (opioids, cannabinoids, glutamate and GABA, cholinergic system, etc.) that were not analyzed in this study. Therefore, one direction of future research should be the identification of other potential neurochemical mechanisms involved in tDCS action.

## Author contributions

AZ, LA-M designed research; ER developed software necessary to perform experiments; AZ and ER performed research; JK-Z performed analytic examinations of blood specimens; IR, JM, and LA-M performed examinations of brain tissues; AZ, JK-Z, IR, and JM analyzed data; AZ and LA-M wrote the paper.

### Conflict of interest statement

The authors declare that the research was conducted in the absence of any commercial or financial relationships that could be construed as a potential conflict of interest.
