# Supplementary material for: Repeated Transcranial Direct Current Stimulation Induces Behavioral, Metabolic and Neurochemical Effects in Rats on High-Calorie Diet
Source: Front Behav Neurosci. 2018 Jan 15;11:262. doi: 10.3389/fnbeh.2017.00262 (PMC5775234; doi:10.3389/fnbeh.2017.00262)

Supplementary Material

**Repeated Transcranial Direct Current Stimulation Induces Behavioral, Metabolic And Neurochemical Effects In Rats On High-Calorie Diet**

**running title: Behavioral and neurochemical aspects of tDCS action**

Agata Ziomber^1^*, Eugeniusz Rokita^2^, Jolanta Kaszuba-Zwoinska^1^, Irena Romańska^3^, Jerzy Michaluk^3^, Lucyna Antkiewicz-Michaluk^3^

^1^ Chair of Pathophysiology, Faculty of Medicine, Jagiellonian University Medical College, Krakow, Poland

^2^ Department of Biophysics, Chair of Physiology, Faculty of Medicine, Jagiellonian University Medical College, Krakow, Poland

^3^ Institute of Pharmacology Polish Academy of Sciences, Department of Neurochemistry, Krakow, Poland

*** - Corresponding author:** [agata.ziomber@uj.edu.pl](mailto:agata.ziomber@uj.edu.pl)

**Supplement 1 (S1).** The effects of high-calorie diet (Ob vs L) and stimulation procedure (Sh vs A vs C) on serum total cholesterol (TCh) [mmol/L], LDL [mmol/L], HDL [mmol/L] and triglicerydes (Tg) [mmol/L]. L - lean intact (n=11); Ob - obese intact (n=10); Sh - obese with sham stimulation (n=11), A– obese with anodal stimulation of the right prefrontal cortex (n=14); C– obese with cathodal stimulation of the left prefrontal cortex (n=11). Data are presented as the mean ± SD. ***P < 0.001 vs L. One-way Anova followed by the Tukey post-hoc test or Kruskal-Wallis test followed by the Dunn post-hoc test (HDL) was used.


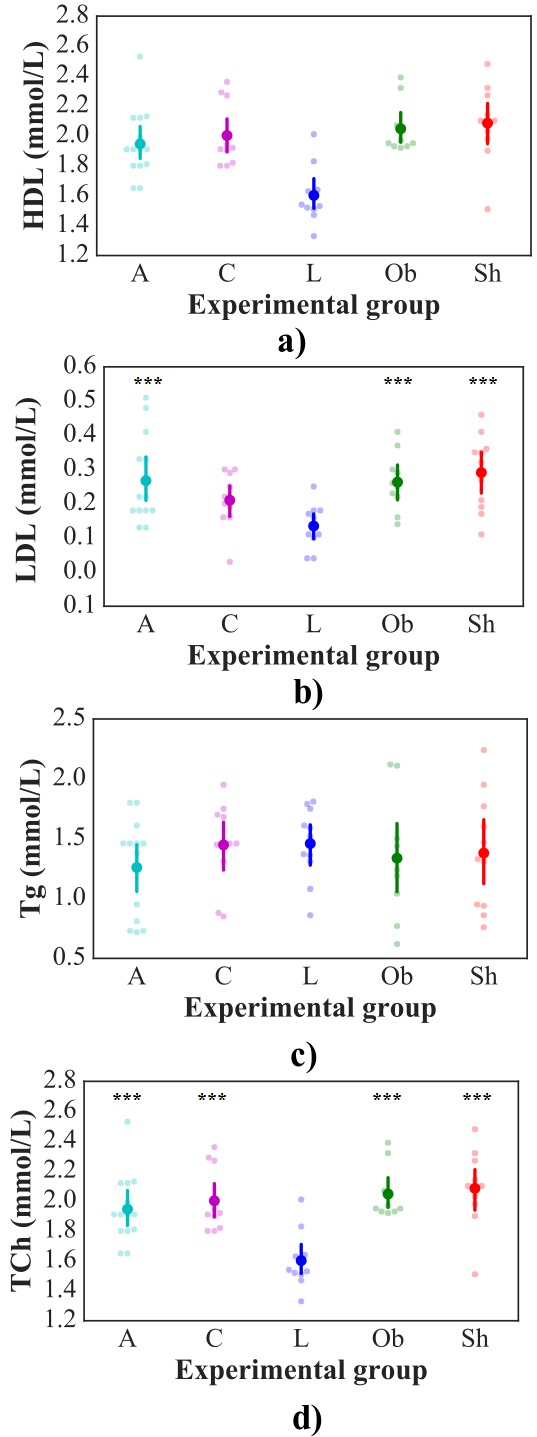


**Supplement 2 (S2).** The effects of high-calorie diet (Ob vs L) and stimulation procedure (Sh vs A vs C) on serum ghrelin [ng/mL] (a) and leptin [ng/mL] (b) concentrations. L - lean intact (n=11); Ob - obese intact (n=10); Sh - obese with sham stimulation (n=11), A– obese with anodal stimulation of the right prefrontal cortex (n=14); C– obese with cathodal stimulation of the left prefrontal cortex (n=11). Data are presented as the mean ± SD. ^T^P < 0.1 vs L; ***P < 0.001 vs L; ^&^ P < 0.05 vs A and C; ^&&^P < 0.01 vs A and C . One-way Anova followed by the Tukey post-hoc test was used.


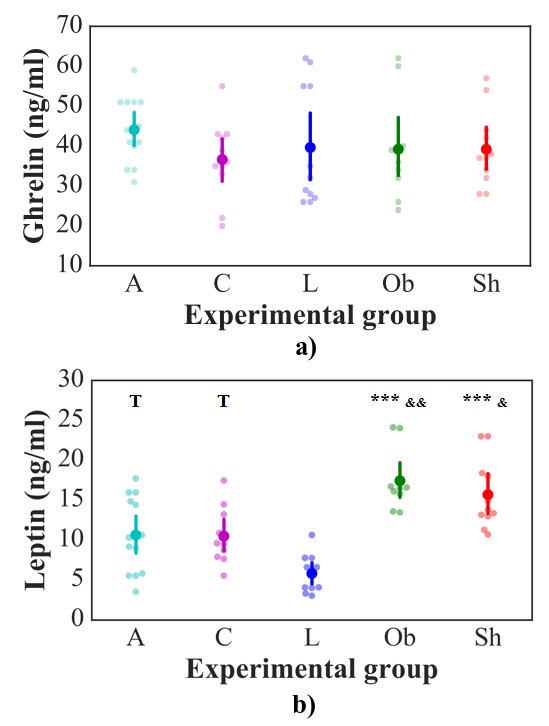


**Supplement 3 (S3).** Pearson’s or Spearman’s correlation between serum HDL [mmol/L], LDL [mmol/L], total cholesterol (TCh) [mmol/L], and triglycerides (Tg) [mmol/L] levels. Positive relationships between TCh and LDL, TCh and HDL as well as LDL and HDL were recorded. Contrary, Tg negatively correlated with other parameters. On the diagonal of the multiple pairwise bivariate distribution plot, the kernel density estimate of the probability density function is shown for each variable subjected to the correlation analysis.


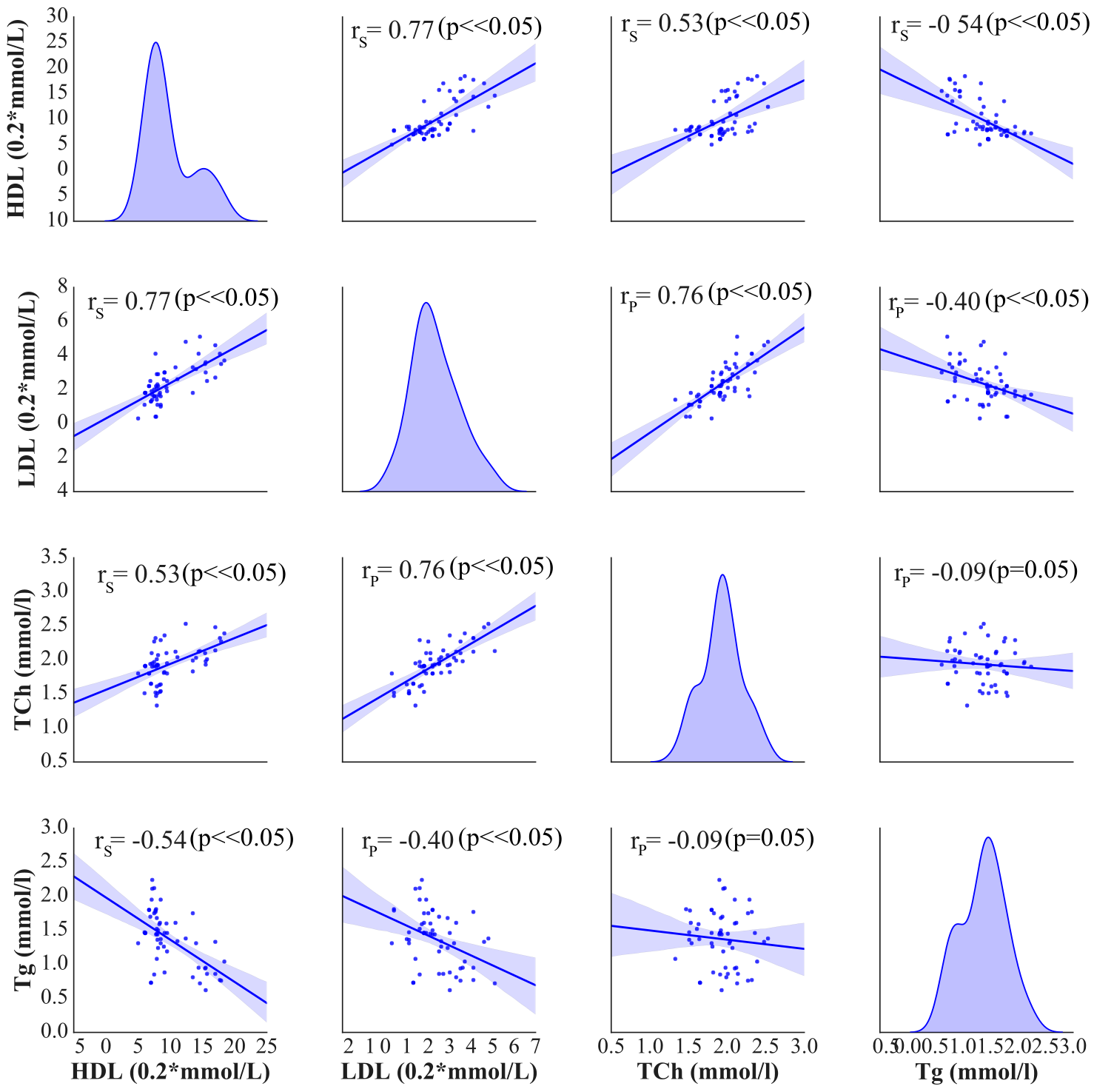


**Supplement 4 (S4).** Brain concentration of monoamines (DA and 5-HT [ng/g tissue]), their metabolites (3-MT, HVA, DOPAC; 5-HIAA [ng/g tissue] and the rate of metabolism (3-MT/DA, HVA/DA, 5-HIAA/5-HT [%]) investigated in FRONTAL CORTEX (FCx), HYPOTHALAMUS (Hyp) and DORSAL STRIATUM (Str) in L - lean intact (n=8) and Ob - obese intact rats (n=8). Data are presented as the mean ± SD. Student T-test was used. No differences were detected between the examined groups (P>0.05).


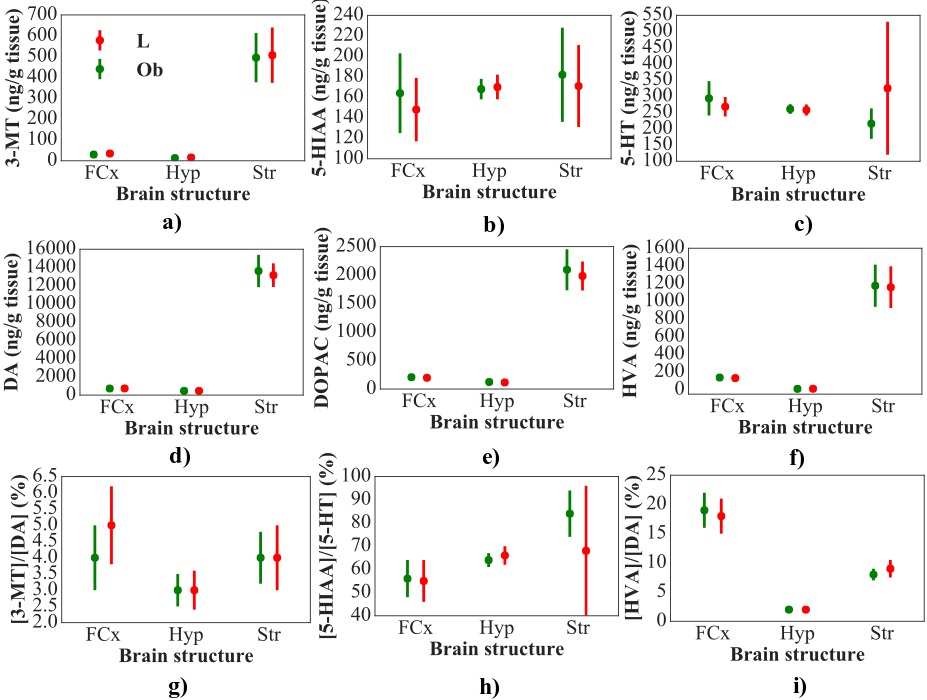


**Supplement 5 (S5).** The effect of repetitive anodal (A) and cathodal (C) tDCS on the brain concentration of monoamines (DA and 5-HT [ng/g tissue]) and their metabolites (3-MT, HVA, DOPAC; 5-HIAA [ng/g tissue] and on the rate of metabolism (3-MT/DA, HVA/DA, 5-HIAA/5-HT [%]) at different time point after the last stimulation investigated in the right and left FRONTAL CORTEX (FCx). A– obese with anodal stimulation of the right prefrontal cortex; C– obese with cathodal stimulation of the left prefrontal cortex; SH - obese with sham stimulation. The number indicates the time (h) from the last stimulation to the brain removal while R (right) or L (left) - the side of the brain examined (i.e. A1R – the rats with anodal stimulation and brain removal 1 hour from the last tDCS, right side of the brain examined). 4 rats per group. Data are presented as the mean ± SD. One-way Anova followed by the Duncan post-hoc test was used. *P < 0.05 vs sham stimulation (SH); **P < 0.01 vs SH.

**
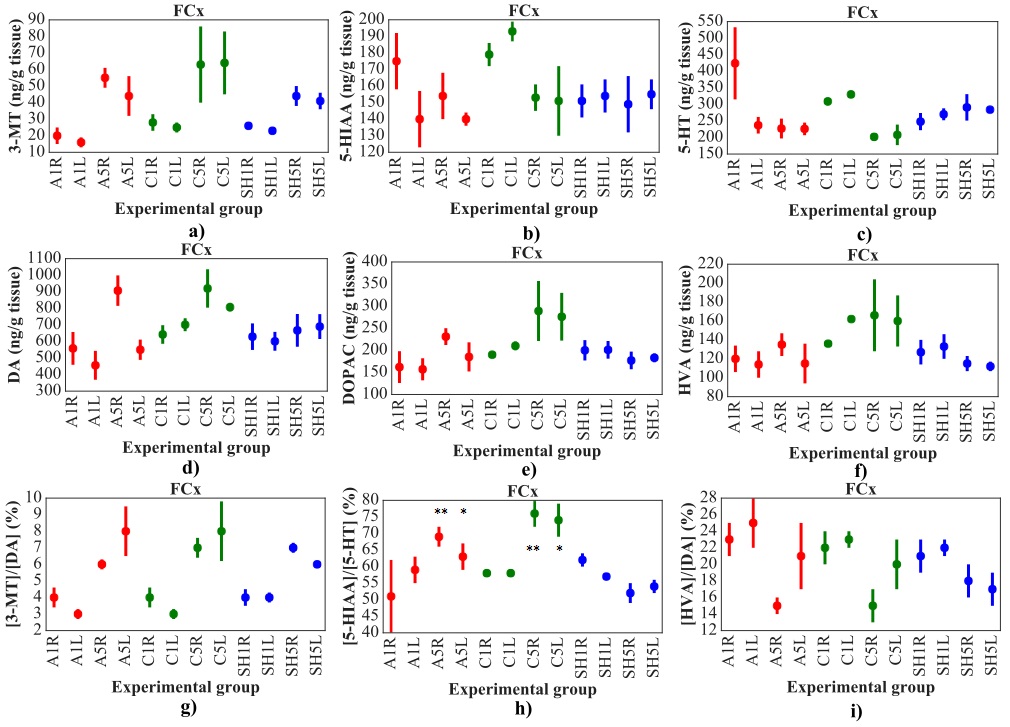
**

**Supplement 6 (S6).** The effect of repetitive anodal (A) and cathodal (C) tDCS on the brain concentration of monoamines (DA and 5-HT [ng/g tissue]) and their metabolites (3-MT, HVA, DOPAC; 5-HIAA [ng/g tissue] and on the rate of metabolism (3-MT/DA, HVA/DA, 5-HIAA/5-HT [%]) at different time point after the last stimulation investigated in the right and left DORSAL STRIATUM (Str). A– obese with anodal stimulation of the right prefrontal cortex; C– obese with cathodal stimulation of the left prefrontal cortex; SH - obese with sham stimulation. The number indicates the time (h) from the last stimulation to the brain removal while R (right) or L (left) - the side of the brain examined (i.e. A1R – the rats with anodal stimulation and brain removal 1 hour from the last tDCS, right side of the brain examined). 4 rats per group. Data are presented as the mean ± SD; One-way Anova followed by the Duncan post-hoc test was used. *P < 0.05 vs sham stimulation (SH); **P < 0.01 vs SH.


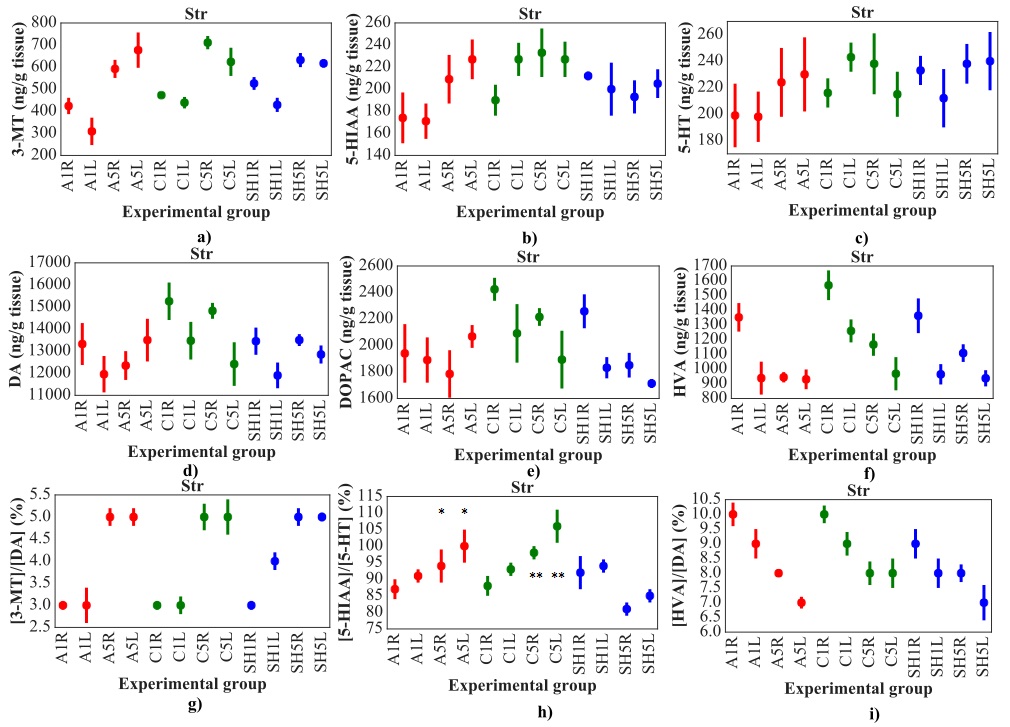


**Supplement 7 (S7).** The effect of repetitive anodal (A) and cathodal (C) tDCS on the brain concentration of monoamines (DA and 5-HT [ng/g tissue]) and their metabolites (3-MT, HVA, DOPAC; 5-HIAA [ng/g tissue] and on the rate of metabolism (3-MT/DA, HVA/DA, 5-HIAA/5-HT [%]) at different time point after the last stimulation investigated in NUCLEUS ACCUMBENS (NAc). A– obese with anodal stimulation of the right prefrontal cortex; C– obese with cathodal stimulation of the left prefrontal cortex; SH - obese with sham stimulation. 4 rats per group. Data are presented as the mean ± SD. One-way Anova followed by the Duncan post-hoc test was used. *P < 0.05 vs sham stimulation (SH).


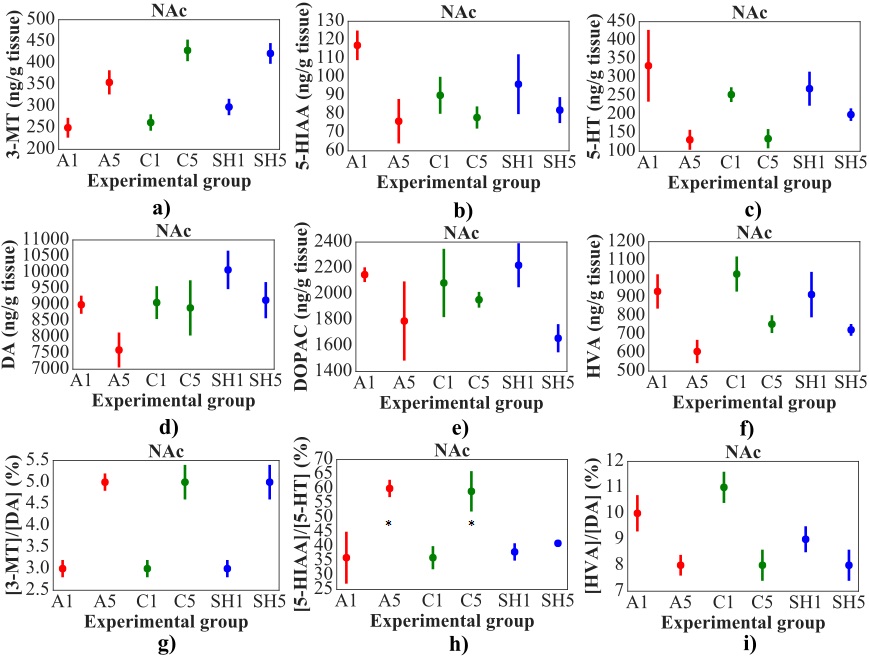


**Supplement 8 (S8).** The effect of repetitive anodal (A) and cathodal (C) tDCS on the brain concentration of monoamines (DA and 5-HT [ng/g tissue]) and their metabolites (3-MT, HVA, DOPAC; 5-HIAA [ng/g tissue] and on the rate of metabolism (3-MT/DA, HVA/DA, 5-HIAA/5-HT [%]) at different time point after the last stimulation investigated in HYPOTHALAMUS (Hyp). A– obese with anodal stimulation of the right prefrontal cortex; C– obese with cathodal stimulation of the left prefrontal cortex; SH - obese with sham stimulation. 4 rats per group. Data are presented as the mean ± SD. One-way Anova followed, when appropriate, by the Duncan post-hoc test was used. No differences between the tested groups were recorded (P > 0.05).

**
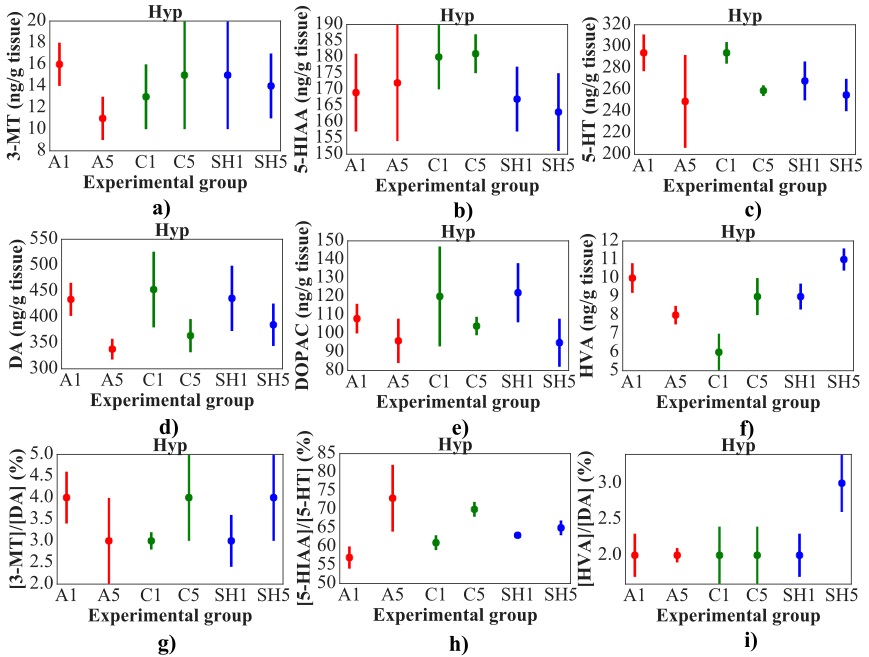
**

**Supplement 9 (S9).** Microscopic illustration of coronar section of the brain exposed to active tDCS application. The frame indicates the area located just below the electrode for tDCS and its magnification beside. No signs of neurotrauma, edema, hematoma of other pathological changes were detected by light microscopy in rats after anodal tDCS of the right prefrontal cortex or cathodal tDCS of the left prefrontal cortex. The bar below the coronar section of the brain (left side) indicates 3.5mm, while this located beneath the magnified brain area - 0.14mm.


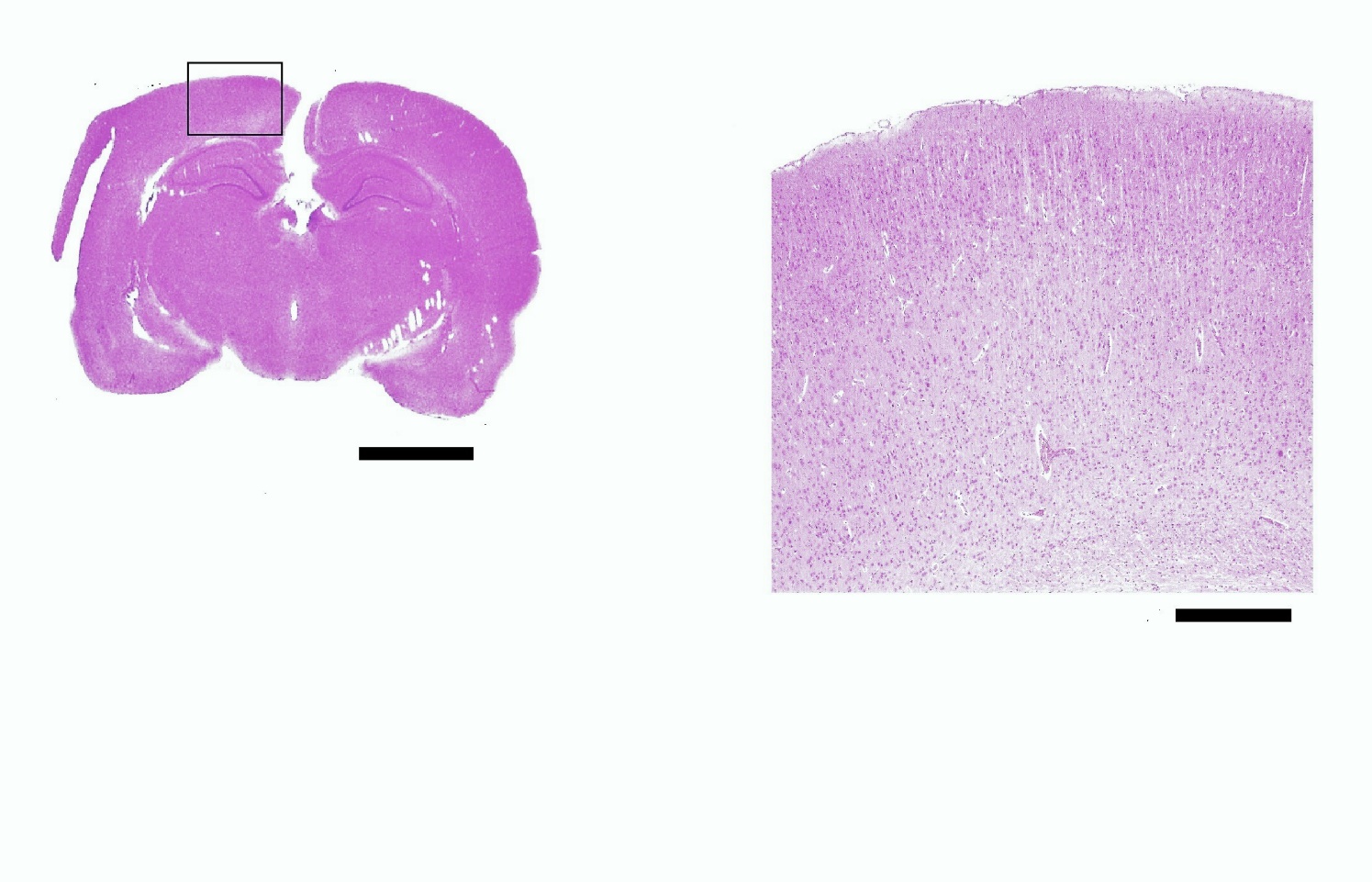

Supplement: Supplementary file 1 [file DataSheet1.docx]
